# Allogeneic DNT cell therapy synergizes with T cells to promote anti-leukemic activities while suppressing GvHD

**DOI:** 10.1186/s13046-024-03247-w

**Published:** 2025-01-28

**Authors:** Jongbok Lee, Hyeonjeong Kang, Branson Chen, Yoosu Na, Ismat Khatri, Fraser Soares, Housheng Hansen He, Arjun D. Law, Tianzhong Pan, Armin Gerbitz, Xiaoyu Zhu, Mark D. Minden, Li Zhang

**Affiliations:** 1 Toronto General Hospital Research Institute, University Health Network, Toronto, ON Canada; 2 Department of Biochemistry and Molecular Biology, University of Calgary, Calgary, AB Canada; 3 Department of Laboratory Medicine and Pathobiology, University of Toronto, Toronto, ON Canada; 4 Princess Margaret Cancer Centre, University Health Network, Toronto, ON Canada; 5 Department of Medical Biophysics, University of Toronto, Toronto, ON Canada; 6 Department of Hematology, the First Affiliated Hospital of USTC, Division of Life Sciences and Medicine, University of Science and Technology of China, Hefei, China; 7 Blood and Cell Therapy Institute, Division of Life Sciences and Medicine, University of Science and Technology of China, Hefei, China; 8 Department of Immunology, University of Toronto, Toronto, ON Canada

**Keywords:** Double negative T cell, Allogeneic hematopoietic stem cell transplantation, Graft-versus-host disease, Graft-versus-leukemia, Donor lymphocyte infusion

## Abstract

Allogeneic hematopoietic stem cell transplantation (allo-HSCT) is a second-line treatment with curative potential for leukemia patients. However, the prognosis of allo-HSCT patients with disease relapse or graft-versus-host disease (GvHD) is poor. CD4^+^ or CD8^+^ conventional T (Tconv) cells are critically involved in mediating anti-leukemic immune responses to prevent relapse and detrimental GvHD. Hence, treatment for one increases the risk of the other. Thus, therapeutic strategies that can address relapse and GvHD are considered the Holy Grail of allo-HSCT. CD3^+^CD4^−^CD8^−^ double-negative T cells (DNTs) are unconventional mature T cells with potent anti-leukemia effects with “off-the-shelf” potential. A phase I clinical trial demonstrated the feasibility, safety, and potential efficacy of allogeneic DNT therapy for patients with relapsing acute myeloid leukemia (AML) post-allo-HSCT. Here, we studied the impact of DNTs on the anti-leukemic and GvHD-inducing activities of Tconv cells. DNTs synergized with Tconv cells to mediate superior anti-leukemic activity. Mechanistically, DNTs released soluble factors which activated and evoked potent anti-leukemic activities of Tconv cells. In contrast, DNTs suppressed GvHD-inducing activities of Tconv cells in a CD18-dependent manner by mediating cytotoxicity against proliferative Tconv cells. The seemingly opposite immunological activities of DNTs were dictated by the presence or absence of AML cells. Collectively, these results support the potential of DNTs as an adjuvant to allo-HSCT to address both disease relapse and GvHD.

**Supplementary Information:**

The online version contains supplementary material available at 10.1186/s13046-024-03247-w.

## Key points

DNTs enhance T cells’ anti-leukemic activity in the presence of AML cells, while suppressing T cells to inhibit GVHD in the absence of AML cells.

**Supplementary Information:**

The online version contains supplementary material available at 10.1186/s13046-024-03247-w.

## Introduction

Allogeneic hematopoietic stem cell transplantation (allo-HSCT) is a curative treatment for patients with hematological malignancies such as high-risk acute myeloid leukemia (AML) [1]. Its therapeutic benefit arises from donor-derived T cell-mediated graft-versus-leukemia (GvL) effect targeting residual leukemic blasts that persist after chemotherapy [2–4]. For relapsing or high-risk hematological malignancy patients, donor lymphocyte infusion (DLI) can be given after allo-HSCT to further augment the GvL effect. Nevertheless, disease relapse remains one of the major causes of mortality in allo-HSCT patients [5]. Furthermore, allo-HSCT and DLI pose the risk of acute GvHD, the leading cause of non-relapse mortality for allo-HSCT patients [6]. Therefore, post-transplant strategies that reduce the chance of relapse while preventing GvHD are considered the Holy-Grail for allo-HSCT recipients [7].

Off-the-shelf allogeneic T cell therapy is considered the next generation of adoptive cellular therapy (ACT) [8–13]. However, the risk of GvHD and host-versus-graft (HvG) rejection limits the safety and efficacy of allogeneic T cell therapy [14]. Recently, our team has developed a novel form of ACT using a subset of T cells defined as CD3^+^ CD4^−^ CD8^−^ double negative T cells (DNTs), which expresses either αβ- or γδ- T cell receptor (TCR). Ex vivo expanded healthy donor-derived allogeneic DNTs induce potent cytotoxic activity against AML and other hematological malignancies in vitro and in xenograft models without causing GvHD [15–17]. We recently completed the first-in-human phase I clinical trial to treat AML patients who relapsed after allo-HSCT with third-party donor-derived DNTs [18]. None of the treated patients showed any signs of GvHD, neurotoxicity, or any other treatment-associated adverse event higher than grade 2. DNT-treated patients trends for improved 1-year survival than the historical controls. Collectively, the trial demonstrated the feasibility, safety, and early signals of the efficacy of allogeneic DNT therapy as a new treatment of relapsed AML post-allo-HSCT.

Interestingly, allogeneic DNTs fulfill the requirements of an off-the-shelf cellular therapy without genetic modifications [19] as they can be obtained from a broad spectrum of donors with minimal restrictions, do not induce off-tumor toxicities including GvHD, function in an MHC-unrestricted manner, and can be cryopreserved. Notably, healthy donor-derived DNTs co-engraft with allogeneic CD4^+^ or CD8^+^ Tconv cells and persist in vivo without signs of rejection [19], suggesting that DNTs may evade the alloreactivity of Tconv cells.

However, prior studies were conducted using in vitro systems or xenograft models that lack Tconv cells, which play a crucial role in determining patient outcomes, particularly in allo-HSCT patients. Thus, the impact of DNT therapy on nearby Tconv cells, especially regarding their anti-leukemic and GvHD-inducing activities, has not been thoroughly studied to date.

This study demonstrates that DNTs and Tconv cells can synergize to promote leukemia clearance in AML-xenograft models. DNTs induced the anti-leukemic response of CD8^+^ T cells by releasing soluble factors upon encountering AML cells. Importantly, DNTs can also suppress the proliferation and effector activity of Tconv cells against alloantigens, thus attenuating GvHD. The opposing immunological activities of DNTs appear to be dictated by the presence or absence of AML cells, where the immunosuppressive activities of DNTs were diminished after encountering AML cells. Collectively, these findings elucidate the mechanisms involved in DNT-mediated GvL effect and GvHD suppression and support using DNTs as an adjuvant in allo-HSCT to address the two leading causes of mortality.

## Results

### DNTs synergize with Tconv cells to mediate superior anti-leukemic activity

Despite the long-term curative potential of allo-HSCT, disease relapse remains the major cause of death in AML patients, particularly those resistant to standard chemotherapy. Ex vivo expanded DNTs can express either TCRγδ^+^ or TCRαβ^+^. Within TCRγδ, DNTs can be Vδ1-TCR^+^, Vδ2-TCR^+^, or Vδ1^−^Vδ2^−^ (Figure S1A). We previously demonstrated that DNT express an array of NK-related molecules like NKG2D, DNAM-1, NKp30, and CD94 [19]. Furthermore, significantly higher expression of KIR genes were observed in DNTs, compared to Tconv cells expanded from the same donor in parallel (Figure S1B), and protein expression of KIR2DL2, KIR2DL3, KIR2DL4, and KIR2DS4 was detected (Figure S1C). These ex vivo expanded DNT mediate potent anti-leukemic activity against AML cells, whereas Tconv cells expanded in parallel do not (Figure S2) and showed no signs of off-tumor toxicities [19, 20].

**Fig. 1:**
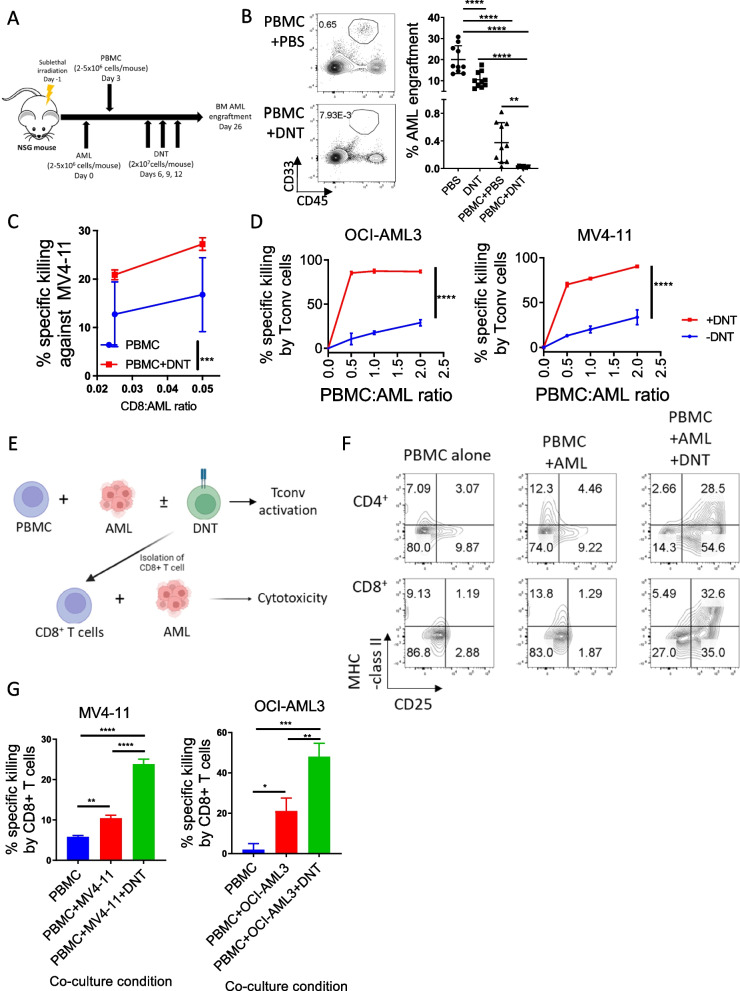
DNTs promote anti-leukemic activity of Tconv cells. **A**, **B** Schematic of xenograft model used to assess the effect of DNTs on GVL (**A**). Sublethally-irradiated NSG mice were systemically administered with human AML cell line, MV4-11, followed by treatment with PBS or human PBMC 3 days post-leukemic cell infusion. Subsequently, mice were treated with ex vivo expanded DNTs or PBS on days 6, 9, and 12 post-leukemia infusion, and bone marrow AML engraftment was determined 26 days post-AML infusion. Flow plots show the representative bone marrow engraftment of human CD45^+^ CD33^+^ MV4-11 cells in mice treated with PBS, DNTs, PBMC + PBS or PBMC + DNT. Dot plots show the AML bone marrow engraftment with each dot representing an individual mouse and horizontal bar representing the mean. The results are a summary of 2 independent experiments with *n* = 10 for each group**.** One-way ANOVA test was used for statistical analysis (**B**). Horizontal bar represents the mean of BM AML engraftment level normalized to PBS control group, each symbol represents individual mouse, and error bars represent SEM. **C** The anti-leukemic activity of CD8^+^ T cells isolated from PBMC + PBS and PBMC + DNT group were compared in an ex vivo killing assay against the initial AML cells, MV4-11, used for engraftment. The cells were incubated at the indicated CD8^+^ T cell-to-MV4-11 cell ratio for 4-h. The result shown is representative of 3 independent experiments done using T cells from 3 different donors. The symbols represent the mean and the error bars represent SD. **D** AML cell lines, OCI-AML3 (left) and MV4-11 (right), were cultured with PBMC-derived Tconv cells at the indicated ratio in the presence or absence of DNTs at 0.2:1 DNT:AML ratio. AML cells alone or AML cells with DNTs at 0.2:1 ratio were used as controls to account for baseline AML cell death. After an overnight incubation, the percent specific killing of AML cells by Tconv cells in the presence or absence DNTs were determined by flow cytometry. The experiments were done in triplicates and the results shown are representative of two independent experiments for each AML cell line. **E**–**G** Schematic diagram of in vitro experiments assessing the impact of DNTs on the anti-leukemic activity of CD8^+^ T cells **E.** Expression of activation markers CD25 and MHC-class II on CD4^+^ and CD8^+^ T cells measured by flow cytometry are shown. The result shown is representative of two independent experiments done using two PBMC-DNT pairs **F**. PBMCs were cultured alone or with MV4-11 cells or MV4-11 cells + DNTs for 3 days. Subsequently, CD8^+^ T cells were isolated and used as effector cells against MV4-11 cells in subsequent 4-h in vitro cytotoxicity assays at 1-to-1 CD8^+^ T cell-to-MV4-11 cell ratio. % specific killing of MV4-11 cells by CD8^+^ T cells from each group are shown. The experiments were done in triplicates, and the result shown is representative of three independent experiments conducted using three PBMC-DNT pairs. One-way ANOVA test was used for statistical analysis **G**. *p < 0.05; **p < 0.01; ***p < 0.001; ****p < 0.0001

Despite extensive study on direct anti-leukemic activity of DNT, their impact on nearby T cell activity has not been studied previously. Here, we investigated whether allogeneic DNTs could further enhance the anti-leukemic effect of Tconv cells. NSG mice infused with a human AML cell line, MV4-11, were treated with PBS, ex vivo expanded DNTs, human PBMC + PBS, or PBMC + DNTs, and the levels of AML in the bone marrow (BM) were assessed 2-weeks post DNT-infusion (Fig. 1A). As previously reported [17, 19], DNTs alone significantly reduced AML levels in the BM (Fig. 1B). A strong anti-leukemia response in PBMC + PBS group was observed, although residual leukemic cells were detected in 9 out of 10 mice by flow cytometry (Fig. 1B). In contrast, co-treating mice with PBMC and DNTs eradicated the disease to below the level of detection by flow cytometry in all mice of the treatment group (Fig. 1B). We observed that both PBMC + PBS and PBMC + DNT treatment significantly reduced the BM AML level in three patient-derived xenograft (PDX) models using AML primary blasts from patients (Figure S3A). Summarizing the BM AML cell engraftment relative to PBS-treated mice across the three PDX models showed a trend towards reduced AML levels was observed in the PBMC + DNT group compared to the PBMC + PBS group, although the difference did not reach statistical significance (*p* = 0.0645) (Figure S3B), potentially due to the limited sample size, variability among patient samples, and the potent anti-leukemic activity mediated by PBMCs alone.

To determine the durability of the response seen in the PBMC + DNT group, the AML engraftment in PBMC + DNT treated mice was compared on early (day 26) or late time points (humane endpoint or day 52). We did not detect AML cells in the BM at these time points, suggesting that mice did not die of leukemia relapse (Figure S4A). In contrast, we detected human CD4^+^/CD8^+^ T cell engraftment in the BM and hair loss observed in mice that survived until the end point, suggesting that the mice died of GvHD (Figure S4B).

To determine whether the superior leukemia control in PBMC + DNT group is due to an additive effect of the endogenous anti-leukemic activities of CD8^+^ Tconv cells, the major anti-leukemia effector cells in PBMC, and DNTs or a synergy between these two cell types, CD8^+^ T cells were isolated from PBMC + PBS and PBMC + DNT treated mice and used as effector cells against the same leukemic cell line (MV4-11) for the AML-xenograft model. CD8^+^ T cells from PBMC + DNT group induced superior cytotoxicity against the AML cells ex vivo than those obtained from PBMC + PBS group (Fig. 1C), suggesting that DNTs promote the anti-leukemic function of CD8^+^ Tconv cells. To further assess this, we compared the viable AML cell counts when AML cell lines (MV4-11 and OCI-AML3) were cultured alone or with PBMC in the presence or absence of a low number of DNTs (0.2:1 DNT-to-PBMC ratio). We observed significantly greater cytotoxicity mediated by Tconv cells against AML cell lines in PBMC + DNT group than PBMC alone group for both cell lines (Fig. 1D). Next, the activation status and anti-leukemic activity of Tconv cells were compared after culturing PBMCs alone or with DNTs, AML cells, or AML + DNT cells for 5 days (Fig. 1E)*.* CD4^+^ and CD8^+^ Tconv cells were preferentially activated when cultured with AML cells and DNTs, but not with AML cells alone, as evidenced by the elevated expression of activation markers CD25 and MHC class II (Fig. 1F). Next, the anti-leukemic activity of CD8^+^ Tconv cells was isolated from these co-cultures against the same AML cells used for the cultures. CD8^+^ Tconv cells obtained from PBMC + AML + DNT co-culture were more cytotoxic against both AML cell lines tested than those obtained from PBMC + AML (Fig. 1G). These results show that DNTs promote the anti-leukemic activity of Tconv cells to yield superior leukemia control.

### Soluble factors secreted by DNT induce anti-leukemic activity of CD8^+^ Tconv cells

**Fig. 2:**
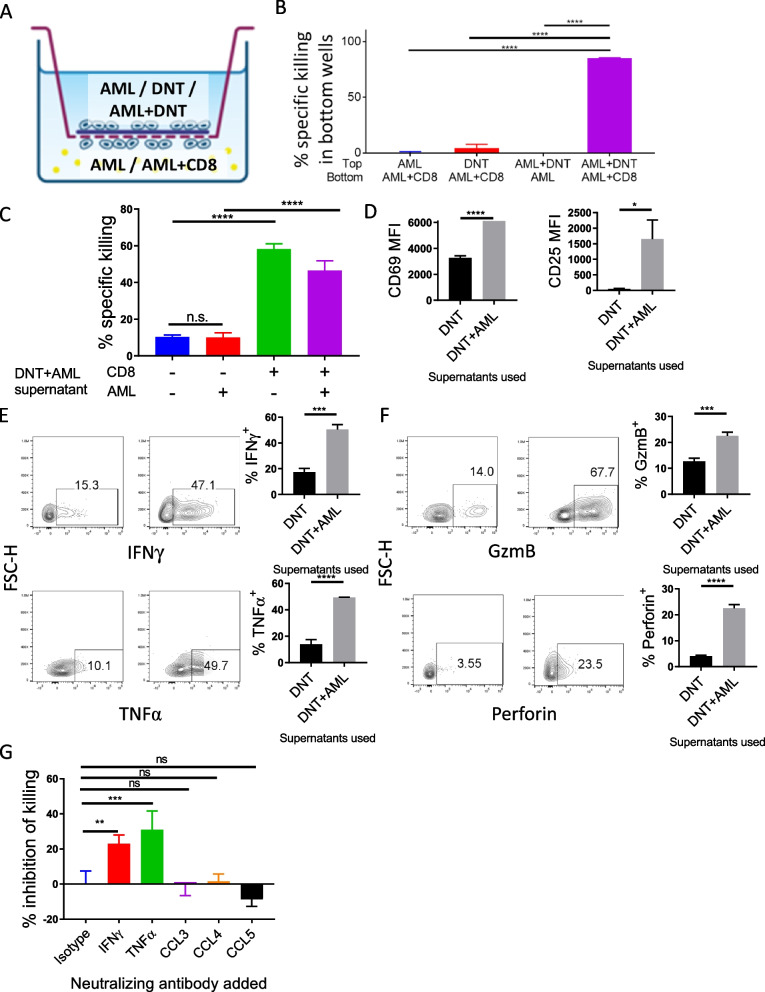
DNTs promote the anti-leukemic activities of Tconv cells through releasing soluble factors. **A** and **B** Schematic diagram of transwell assays conducted to assess the role of soluble factors in DNT-induced anti-leukemic activity of CD8^+^ T cells (**A**). AML (MV4-11), DNTs, or AML + DNTs were seeded in the top wells and AML or AML + CD8^+^ T cells were seeded in the bottom wells, separated by a permeable membrane. % specific killing of AML cells in the bottom wells were determined by flow cytometry. Experiments were conducted in triplicates and the result shown is representative of two independent experiments. One-way ANOVA test was used for statistical analysis. ****p < 0.0001. **C** CD8^+^ T cells, MV4-11 cells, or both were treated with supernatants taken from DNT + MV4-11 co-culture (^DNT+AML^supernatant). Subsequently, the cells were cocultured at 1:1 effector-to-target ratio for 4-h. % specific killing of AML cells by CD8^+^ T cells are shown. Experiments were conducted in triplicates and the result shown is representative of three independent experiments. One-way ANOVA test was used for statistical analysis. ****p < 0.0001. **D-F** CD8^+^ T cells treated with ^DNT+AML^supernatant or ^DNT^supernatant for 1 day were cultured with MV4-11 cells. Subsequently, the expression of activation markers, CD69 and CD25 (**D**), inflammatory cytokines, IFNγ and TNFα (**E**), and effector molecules, granzyme B (gzmB) and perforin (**F**), by CD8^+^ T cells were determined by flow cytometry. Flow plots show the representative result. The bar graphs show the average MFI (**C**) or % expressed (**D** and **E**) from three separate experiments ± SEM. *Student’s t-test* was used for statistical analysis. **G** CD8^+^ T cells cultured with MV4-11 cells in ^DNT+AML^supernatant in the presence of blocking antibodies against IFNγ, TNFα, CCL3, CCL4, or CCL5 or an isotype control. Subsequently, % inhibition of CD8^+^ T cell mediated killing against MV4-11 cells was determined by flow cytometry. The experiments were done in triplicates and the result shown is representative of three independent experiments. One-way ANOVA test was used for statistical analysis. **p < 0.01; ***p < 0.001

To elucidate the mechanism of how DNTs promote the anti-leukemic activity of Tconv cells, we first assessed whether this phenomenon is through a contact-dependent or -independent mechanism using transwell assays as described in Fig. 2A. Culturing DNT or AML cells in the top well did not affect the viability of AML cells cultured with CD8^+^ Tconv cells in the bottom well. Similarly, seeding DNTs and AML cells in the top well did not affect the viability of AML cells cultured alone in the bottom wells. In contrast, there was a significant degree of cytotoxicity aginst AML cells when DNTs were cultured with AML cells in the top well and CD8^+^ Tconv cells incubated with AML cells in the bottom well (Fig. 2B). This suggests that DNTs promote the anti-leukemic activity of Tconv cells through the release of soluble factors.

To determine whether the soluble factors secreted by DNTs upon encountering AML cells sensitize AML cells to CD8^+^ Tconv cell-mediated cytotoxicity or induce the anti-leukemic activity of CD8^+^ Tconv cells, AML cells, CD8^+^ Tconv cells or both were cultured in supernatants taken from DNT alone culture (^DNT^supernatant) or DNT + AML co-culture (^DNT+AML^supernatant) for 24 h. Subsequently, the CD8^+^ Tconv and AML cells were washed and cultured together in fresh media. CD8^+^ Tconv cells pre-cultured in the ^DNT+AML^supernatant induced significant cytotoxicity against AML cells while pre-treating AML cells with ^DNT+AML^supernatant did not affect CD8^+^ Tconv cell-mediated cytotoxicity (Fig. 2C), demonstrating that DNTs promote the anti-leukemic activity of CD8^+^ Tconv cells directly rather than sensitizing AML cells to CD8^+^ Tconv cell-mediated cytotoxicity.

Next, we examined the expression of activation markers, inflammatory cytokines, and effector molecules by CD8^+^ Tconv cells cultured with AML cells in ^DNT^supernatant or ^DNT+AML^supernatant. The expression of CD69 and CD25 were significantly higher on CD8^+^ Tconv cells cultured in ^DNT+AML^supernatant than those cultured in ^DNT^supernatant (Figs. 2D and S2). Similarly, an increased percentage of CD8^+^ Tconv cells expressing IFNγ and TNFα (Fig. 2E), and effector molecules, perforin and granzyme B (Fig. 2F), were detected when CD8^+^ Tconv cells were cultured with MV4-11 cells in ^DNT+AML^supernatant than in ^DNT^supernatant. These findings indicate that the soluble factors produced by DNTs upon encountering AML cells promote activation and effector activities of CD8^+^ Tconv cells.

To identify the soluble factors released by DNTs that induce the anti-leukemic activity of CD8^+^ Tconv cells, the expressions of different inflammatory cytokine and chemokine genes were compared between ex vivo expanded DNTs and Tconv cells from four donors. We observed that *IFNG, TNF, CCL3, CCL4, and CCL5* were consistently expressed at a higher level by DNTs than Tconv cells from the same donors (Figure S5). To investigate the functional relevance of these molecules, CD8^+^ Tconv cells were co-cultured with MV4-11 or AML2 in corresponding ^DNT+AML^supernatant in the presence of neutralizing antibodies to these molecules. We observed that neutralizing TNFα and IFNγ significantly reduced the cytotoxic activity of CD8^+^ T cells against AML cells, while CCL3, CCL4, and CCL5 neutralization had no effect (Fig. 2G). These results demonstrate that encountering AML causes DNTs to release IFNγ and TNFα, which promote the anti-leukemic activity of nearby CD8^+^ Tconv cells.

### DNTs suppress GvHD in xenograft models

**Fig. 3:**
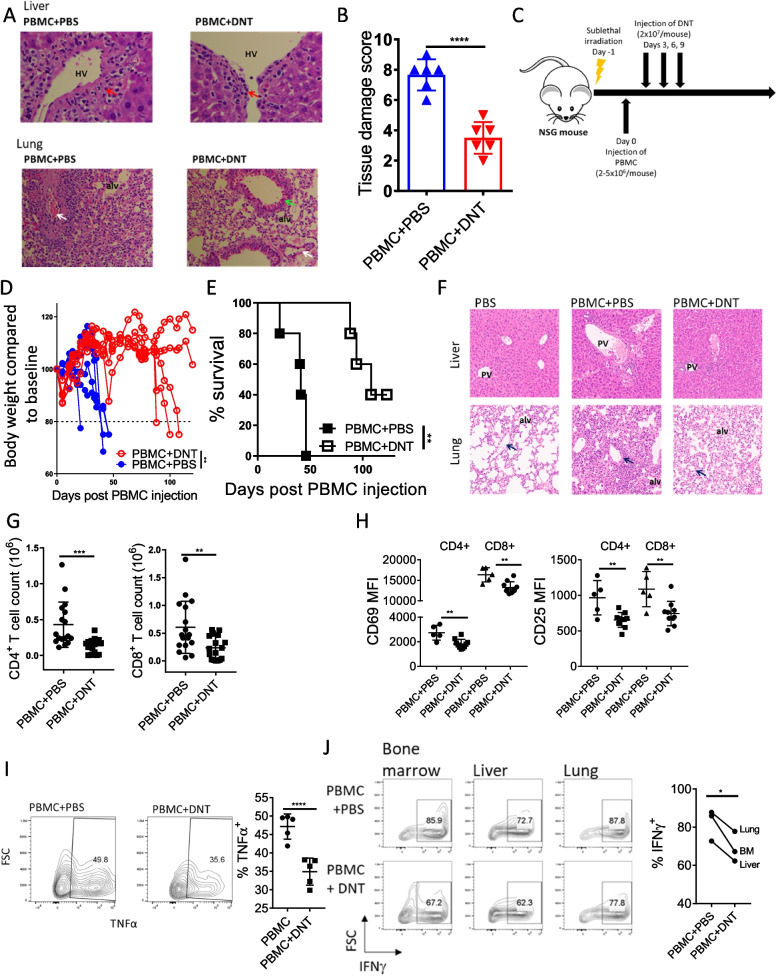
DNTs attenuate GvHD caused by Tconv cells. **A** and **B** NSG mice inoculated with AML cell line MV4-11 were treated with PBMCs + PBS or PBMCs + DNT. On day 26 post-AML injection, liver and lung tissues were formalin-fixed and stained with H&E, as illustrated by the schematic diagram. Representative H&E-stained slides of the liver (400 × magnification) and lung (200 × magnification) from each group are shown. Red arrows indicate the sites of perivenular inflammation, white arrows indicate the vessels, and green arrows indicate bronchioles. HV – hepatic vein; alv – alveoli (**A**). Liver and lung H&E stained slides from PBMC + PBS and PBMC + DNT-treated groups were blindly scored by a pathologist for the degree of tissue damage. Each dot represents a sample, and horizontal bars represent the mean ± SD. Student’s *t*-test was used for statistical analysis (**B**). Data shown are representative of 2 independent experiments. ****p < 0.0001. **C**-**F** Schematic diagram of the PDX model used to determine the GvHD-suppressive activity of DNTs in vivo. Sub-lethally irradiated NSG mice were intravenously injected with 2–5 × 10^6^ human PBMC followed by treatment with ex vivo expanded allogeneic DNTs or PBS on days 3, 6, and 9 post-PBMC infusion (**C**). Mouse body weight was measured twice a week until the end of the study on day 120, and each line represents an individual mouse % body weight change (**D**), and mice survival (**E**) was monitored. Two-way ANOVA test (**D**) and log-rank test (**E**) were used for statistical analysis. On day 28, liver (top) and lung (bottom) tissues were stained with hematoxylin and eosin (H&E) (50 × magnification). Blue arrows indicate the vessels. PV – portal vein; alv – alveoli (**F**). The data shown are representative from each treatment group (PBS, PBMC + PBS, and PBMC + DNT; *n* = 5 per group) and are representative of 3 independent experiments using T cells from 3 different donors. **G** and **H** Total number (**G**) and the expression of activation markers (**H**), CD69 and CD25, on CD4^+^ or CD8^+^ T cells obtained from the liver of leukemia-engrafted mice treated with PBMC + PBS or PBMC + DNT DNT (*n* = 17 for each group for counts; *n* = 5–10 for CD69 and CD25 expression). Each dot represents one mouse, horizontal bars represent the mean, and error bars represent ± SD. The result shown is representative of 2 independent experiments. Student’s *t-*tests was used for statistical analysis. **p < 0.01; ***p < 0.001. **I** Intracellular expression of TNFα on CD8^+^ T cells obtained from the bone marrow of leukemia-engrafted mice treated with PBMC + PBS or PBMC + DNT. Contour flow plots show the representative expression from each treatment group. Each dot represents one mouse, horizontal bars represent the mean, and error bars represent ± SD. The results shown are representative of 3 biological replicates from 2 independent experiments. Student’s *t-*tests were used for statistical analysis. ****p < 0.0001. **J** BM, liver, and lung tissues from each group were pooled (*n* = 5 per group), and Tconv cells were harvested, and ex vivo stimulated with αCD3/CD28 activation beads for 4 h. Subsequently, intracellular expression of IFNγ on the harvested Tconv cells was determined. Each paired dot represents the Tconv cells from lung, BM, or liver. The results shown is representative of 2 biological replicates. Student’s *t-*tests was used for statistical analysis. *p < 0.05

The major side effect of allo-HSCT and DLI is the risk of GvHD. DNTs augument the anti-leukemic response of Tconv cells, and the infusion of xenogeneic or allogeneic DNTs does not induce GvHD in xenograft models [19] and AML patients [20]. However, mice infused with PBMC + DNT eventually developed and died of GvHD. Given that DNTs can enhance the anti-leukemic activity of CD8^+^ Tconv cells, we concurrently studied the impact of DNTs on GvHD-inducing activities of Tconv cells in the AML xenograft model presented in Fig. 1A, by analyzing the liver and lung tissues harvested from the leukemia-bearing mice treated with PBMC + PBS or PBMC + DNT for the degree of tissue damage (Fig. 3A). Despite the superior anti-leukemic activity seen in PBMC + DNT treated mice, histological analysis showed that the tissues from the PBMC + DNT group had less severe signs of GvHD than those in PBMC + PBS treated ones, as evidenced by less perivenular inflammation and mononuclear infiltrates in the liver and lower levels of severe perivascular inflammation with arteritis and septal inflammation in lungs compared to the PBMC + PBS treated group (Fig. 3A). Further, lower tissue damage scores were assigned to the tissue samples from PBMC + DNT group relative to PBMC + PBS group, blindly evaluated by a pathologist (Fig. 3B). This suggested that DNTs can suppress GvHD induced by Tconv cells. To further investigate this, the ability of DNTs to suppress GvHD was directly tested using a previously established xenogeneic GVHD model [21, 22], where naïve-NSG mice were infused with PBMC + PBS or PBMC + DNT. (Fig. 3C). Mice in the PBMC + DNT group showed delayed onset of body weight reduction (Fig. 3D), significantly prolonged survival compared to PBMC + PBS group (Fig. 3E), and less liver and lung tissue damage by histology (Fig. 3F). Consistent with these findings, significantly lower numbers of Tconv cells were obtained from the liver of PBMC + DNT treated mice (Fig. 3G); these Tconv cells showed reduced expression of activation markers CD25 and CD69 (Fig. 3H). To further assess the impact of DNT on effector activities of Tconv cells, we compared the level of inflammatory cytokine production by Tconv cells harvested from the PBMC + PBS group with those obtained from PBMC + DNT group. The ability of DNTs to inhibit Tconv cell activation was further supported by the significantly reduced frequencies of TNFα^+^ and IFNγ^+^ Tconv cells harvested from the PBMC + DNT treated group than those obtained from PBMC + PBS group (Fig. 3I and J). These results demonstrate that ex vivo expanded DNTs effectively suppress GvHD in a xenograft model.

### DNT-mediated suppression of Tconv cells involves cytotoxicity and CD18

**Fig. 4:**
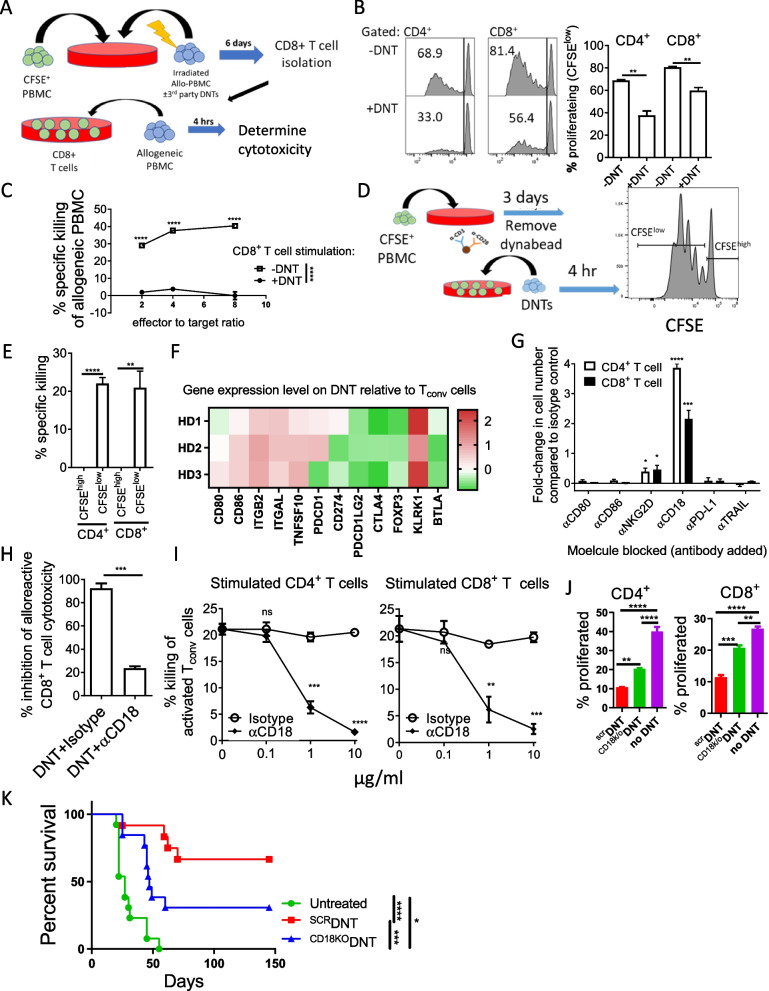
DNTs suppress GvHD in an CD18 dependent manner. **A**-**C** Schematic diagram of mixed-lymphocyte reaction (MLR) assay conducted to assess the impact of DNTs on the alloreactivity of Tconv cells (**A**). CFSE-labelled PBMCs were stimulated with irradiated allo-PBMCs with or without DNTs for 6 days. Proliferation of Tconv cells was determined by CFSE dilution (**B**). After stimulation, CD8^+^ T cells were isolated and used as effector cells against the same allogeneic PBMCs initially used for stimulation. Percent killing of allogeneic CD4^+^ and CD8^+^ T cells by CD8^+^ T cells stimulated in the presence (●) or absence (□) of DNTs at varying effector to target ratio is shown (**C**). The result shown is representative of five independent experiments using 5 different T cell-PBMC pairs. Two-way ANOVA test was used for statistical analysis**.** **p < 0.01. **D** and **E** In vitro cytotoxicity of DNTs against proliferating and non-proliferating T_conv_ cells. CFSE-labeled PBMCs were stimulated with αCD3/αCD28 activation beads for 3 days. After removal of the beads, stimulated Tconv cells were co-cultured with DNTs for 4 h at 4:1 DNT:Tconv ratio (**D**). The bar graph shows the percent specific killing mediated by DNTs against proliferating (CFSE^high^) and non-proliferating (CFSE^low^) CD4^+^ or CD8^+^ T cells **(E)**. The result is representative of 3 independent experiments done using T cells from 3 different donors. Student’s *t-*tests was used for statistical analysis. Error bars represent ± SD. **p < 0.01; ****p < 0.0001. **F** RNAseq to determine the expression levels of genes involved in regulation of immune cell activation on ex vivo expanded DNTs relative to Tconv cells expanded in parallel from the same donors (*n* = 3). **G** CFSE-labeled (1 µM) Tconv cells were stimulated with αCD3/CD28 activation beads in the presence or absence of DNTs. Antibodies against molecules indicated or corresponding isotype control (10 µg/ml) at 4:1 DNT:Tconv cell ratio were added for 5 days. Numbers of CD4^+^ (empty bar) and CD8^+^ (filled bar) T cells at the end of the suppression assays in the presence of various blocking antibody relative to their isotype control were determined. The result shown is mean of 3 independent experiments for each molecule done using T cells from 2–3 different donors. Student’s *t-*tests were used for statistical analysis. *p < 0.05; **p < 0.01; ***p < 0.001; ****p < 0.0001. **H** MLR assays were set up as described in Fig. 4A in the presence of blocking antibody against CD18 (10µg/ml) or corresponding isotype control. The percentage inhibition of alloreactive CD8^+^ T cell activity by DNTs is shown. Student’s *t-*test was used for statistical analysis. ***p < 0.001. **I** In vitro killing assays were conducted using DNTs against activated PBMCs, as described in Fig. 4D, with increasing concentrations of CD18 blocking antibody (filled) or isotype control (empty). % specific killing against CD4^+^ T cells (left) and CD8^+^ T cells (right) are shown. The results shown are representative of 3 independent experiments using T cells from 2 different donors. One-way ANOVA test was used for statistical analysis. **p < 0.01; ***p < 0.001; ****p < 0.0001. **J** In vitro suppression assays were conducted using ^CD18KO^DNTs or ^scr^DNT against Tconv cells stimulated with irradiated allogeneic PBMC at 4:1 DNT to PBMC ratio. % proliferated CD4^+^ (left) and CD8^+^ (right) T cells are shown. The results shown are representative of 2 independent experiments using T cells from 2 different donors. **p < 0.01; ***p < 0.001; ****p < 0.0001. **K** Sub-lethally irradiated NSG mice were intravenously injected with 2 × 10^6^ human PBMC followed by treatment with PBS (*n* = 13), 1.5 × 10^7 scr^DNT (*n* = 12), or 10^7^ 1.5 × ^CD18KO^DNTs (*n* = 13) on days 3 and 6 post-PBMC infusion, and the mice survival was monitored. Log-rank test was used for statistical analysis. The results are pooled from 2 independent experiments using T cells from 2 different donors. *p < 0.05; ***p < 0.001; ****p < 0.0001

To investigate the mechanisms involved in DNT-mediated suppression of GvHD, mixed lymphocyte reaction (MLR) assays were conducted where CFSE-labeled PBMCs were stimulated with allo-antigen derived from irradiated allo-PBMCs in the presence or absence of DNTs. Subsequently, the proliferation of Tconv cells and the cytotoxicity of CD8^+^ Tconv cells against the same allo-PBMCs were assessed (Fig. 4A). DNTs significantly inhibited the proliferation of CD4^+^ and CD8^+^ Tconv cells (Fig. 4B). Further, the CD8^+^ Tconv cells isolated from MLR assay done in the presence of DNTs failed to evoke cytotoxicity against the same allo-PBMC used in the MLR assay in the subsequent cytotoxicity assay, while those stimulated in the absence of DNTs mediated potent alloreactivity (Fig. 4C). These results show that DNTs can prevent the onset of the alloreactivity of Tconv cells.

Inducing apoptosis on activated T cells is one of the mechanisms employed by CD4^+^ T_reg_ cells to mediate immune suppression [23]. Also, DNTs are capable of inducing potent cytotoxicity against leukemic blasts. Hence, we tested whether DNTs induce cytotoxicity against activated Tconv cells to suppress their activity. CFSE-labelled Tconv cells activated with αCD3/αCD28 activation beads for three days were co-cultured with DNTs, and the degree of killing of proliferating (CFSE^low^) and non-proliferating (CFSE^high^) Tconv cells were compared (Fig. 4D). In agreement with the lack of cytotoxicity against naïve PBMCs and normal tissues [17, 19], DNTs did not induce cytotoxicity to CFSE^high^ non-proliferating Tconv cells; in contrast, DNTs mediated a significant degree of cytotoxicity toward CFSE^low^ proliferating Tconv cells (Fig. 4E).

To investigate molecules involved in the regulatory activity of DNTs, DNTs and Tconv cells from the same donors were expanded in parallel and analyzed by RNA sequencing. The expression of genes involved in the regulation of immune cell activation was compared. DNTs showed higher expression of CD86, LFA-1 subunit genes, *ITGB2* and *ITGAL* (encode CD18 and CD11a, respectively), *TNFSF10* (encodes TRAIL) and *KLRK1* (encodes NKG2D) and reduced expression of CD4^+^ FoxP3^+^ regulatory T cell (Treg) associated genes, *PDCD1LG2* (encodes PD-L2), *CTLA4* and *FOXP3*, than Tconv cells in all three donors (Fig. 4F). Relative to Tconv cells expanded in parallel from the same donor, higher levels surface protein expression of CD86, CD18, CD11a, NKG2D, and DNAM-1 on DNTs were confirmed, whereas the membrane TRAIL, PD-L2, CD80 expression levels were low (Figure S6).

To determine the functional relevance of these molecules, blocking antibodies against each molecule or its ligand or corresponding isotype controls were added to the in vitro suppression assays. Blocking CD18 most significantly inhibited DNT-mediated suppression on Tconv cells, resulting in 3.86-folds and 2.16-folds higher numbers of CD4^+^ and CD8^+^ Tconv cells at the end of the suppression assay than in the presence of isotype control (Fig. 4G). Also, blocking CD18 inhibited DNT-mediated suppression of CD8^+^ Tconv cell-mediated cytotoxicity against allo-PBMC (Fig. 4H), and DNT-mediated cytotoxicity against proliferative Tconv cells was blocked by CD18-blocking antibody in a dose-dependent manner (Fig. 4I). Further supporting the involvement of CD18, an increased expression of CD18 ligand, ICAM-1, was observed on activated Tconv cells relative to naïve Tconv cells (Figure S7).

Since CD18 is expressed by both DNT and Tconv cells, CD18 blocking antibodies may have unexpected activity by binding to CD18 expressed on Tconv cells. To that end, CD18 on DNTs was knocked-out (KO) using CRISPR/Cas9 technology (^CD18KO^DNTs; Figure S8), and the inhibitory activity of ^CD18KO^DNT was assessed. PBMCs stimulated with irradiated allogeneic PBMC (Fig. 4J) proliferated significantly better in the presence of ^CD18KO^DNT than the scramble control DNTs (^SCR^DNT). Also, the ability of ^CD18KO^DNT to rescue mice from xenogeneic GvHD was significantly compromised compared to the ^SCR^DNT control (Fig. 4K and Table S1). These results demonstrate the role of CD18 on DNT-mediated suppression of Tconv cell-mediated GvHD.

Next, we assessed the role of CD18 on DNT-induced anti-leukemic activity of CD8^+^ T cells. PBMC were cultured with AML cells in the presence of ^CD18KO^DNT or ^SCR^DNT. Subsequently, the CD8^+^ T cells activated by ^CD18KO^DNT or ^SCR^DNT were isolated and used as effector cells against fresh AML cells to compare their anti-leukemic activity. No differences in the cytotoxicity of CD8^+^ T cells against AML cells were observed (Figure S9A). Similarly, culturing AML cells with ^CD18KO^DNT or ^SCR^DNT with AML cells in the top well and CD8^+^ T cells and AML cells in the bottom well, as done in Fig. 2G, resulted in comparable increase in the cytotoxicity of CD8^+^ T cells against AML cells in the bottom well (Figure S9B), These results suggest that while CD18 is an important molecule for GvHD suppression, it does not affect the ability of DNTs to promote the GvL activity of CD8^+^ T cells.

### Encountering with AML reduced DNT immunosuppressive activity

**Fig. 5:**
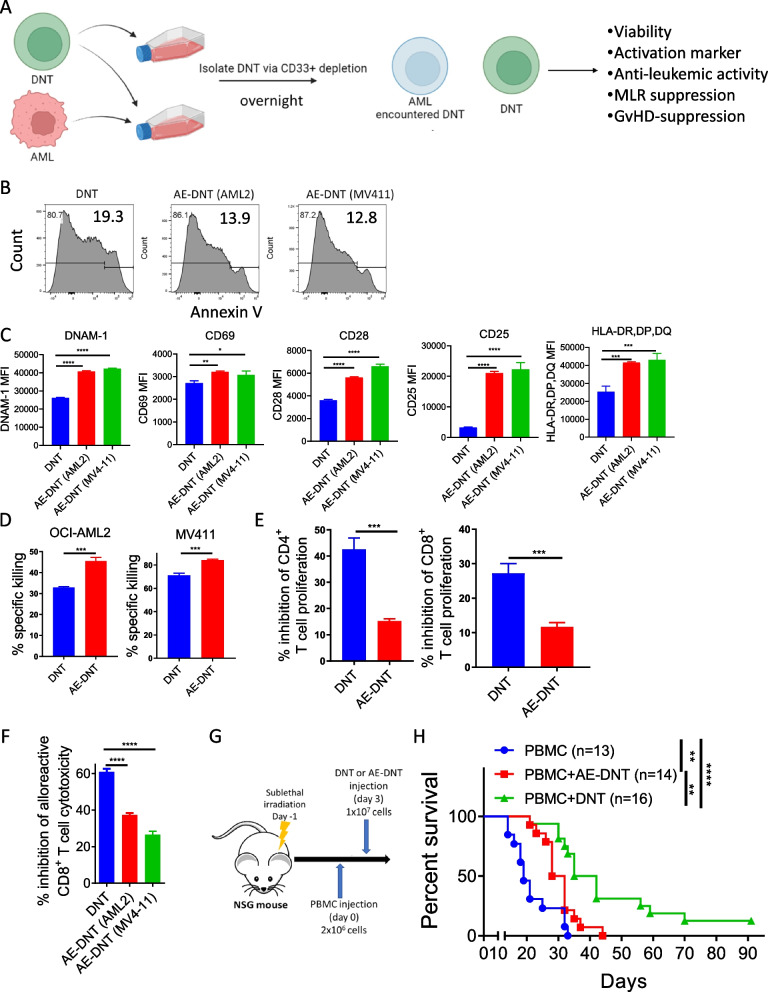
DNTs show reduced immunosuppressive activities in the presence of AML cells. **A**-**F** Schematic diagram of the experiments done to compare the anti-leukemic and immunosuppressive activities of DNTs and AE-DNTs (**A**). DNTs were cultured alone or with AML cell lines, MV4-11 or OCI-AML2, overnight. Subsequently, the viability (**B**), activation markers, DNAM-1, CD69, CD28, CD25, and HLA-DR,DP,DQ, **(C)** of DNT and AE-DNTs, their ability to mediate cytotoxicity against AML cells (**D**), to inhibit proliferation of Tconv cells against irradiated allo-PBMC (**E**), or suppress the alloreactivity of CD8^+^ T cells **(F)** were compared. Each experiment was done in triplicates, and the results shown are representative of three independent experiments. One-way ANOVA (**C** and **F**) or Student’s t-tests (**D** and **E**) were used for statistical analysis. *p < 0.05; **p < 0.01; ***p < 0.001; ****p < 0.0001. **G** and **H** Schematic diagram of GvHD-xenograft model used to assess the GvHD-suppressive activity of AE-DNTs (**G**). Sublethally irradiated NSG mice were infused with 2 × 10^6^ PBMCs alone (*n* = 15) or with 10^7^ DNT (*n* = 15) or AE-DNTs (*n* = 16). Mice survival was monitored. The graph shown is pooled result from three independent experiments. Log-rank test was used for statistical analysis. **p < 0.01; ****p < 0.0001

Microenvironments can dictate the type of immune response mediated by immune cells. A noteworthy difference between GvHD and disease relapse is the major site of disease manifestation. Hence, the seemingly opposing functions of DNTs may be dictated by the microenvironment, where DNTs effectively inhibit Tconv cell alloresponse in the absence of leukemic targets while their immunosuppressive activity is dampened when DNTs are activated in the presence of. To test this hypothesis, DNTs were cultured with or without AML cell lines MV4-11 or OCI-AML2. Subsequently, the viability, anti-leukemic function, and GvHD-suppressive activities of DNTs and AML-encountered DNTs (AE-DNTs) were compared (Fig. 5A). The viability of DNTs and AE-DNTs was comparable (Fig. 5B) but AE-DNTs expressing higher levels of the activating receptor, DNAM-1, and activation markers CD69, CD28, CD25, and HLA-DP, DQ, DR than DNTs (Fig. 5C). Also, AE-DNTs mediated superior cytotoxicity against MV4-11 and OCI-AML2 cells than DNTs (Fig. 5D). However, the ability of AE-DNTs to suppress the proliferation of CD4^+^ or CD8^+^ Tconv cells stimulated with allo-antigen was significantly reduced compared to non-AML experienced DNTs (Fig. 5E). Similarly, the cytotoxicity of CD8^+^ Tconv cells isolated from MLR conducted in the presence of AE-DNTs against allo-PBMC was significantly higher compared to those stimulated with allo-antigen in the presence of DNTs, suggesting that DNTs have a reduced capacity to suppress alloreactive Tconv cells after encountering AML cells (Fig. 5F). To assess the GvHD-suppressive activities of AE-DNTs, mice were infused with human PBMC plus PBS, DNT or AE-DNTs (Fig. 5G). AE-DNTs were significantly less effective at rescuing mice from xenogeneic GvHD than non-AML experienced DNTs as the AE-DNT-treated group had significantly lower survival rate than those treated with DNTs (Fig. 5H). These results support that DNTs can suppress GvHD via immune regulatory activity in the absence of leukemic targets and are able to induce GvL-response of CD8^+^ Tconv cells in the presence of AML cells.

## Discussion

DLI is effective for treating leukemia relapse in allo-HSCT patients but often leads to acute GvHD [24]. Conversely, post-transplant cyclophosphamide, commonly used to prevent or manage GvHD is linked to a higher risk of disease relapse [25]. Current post-transplant interventions either enhance or suppress the overall immune response to address relapse or GvHD, often resulting in a trade-off between controlling GvHD and preventing relapse. Consequently, there is a need for new therapeutic approaches that can effectively address relapse without causing GvHD or, even better, suppressing GvHD induced by donor T cells. However, considering the essential role of donor-derived T cells in both GvL and GvHD, developing a single agent capable of simultaneously promoting and inhibiting T cell responses to prevent relapse and GvHD is challenging, particularly if the strategies are focused on broadly targeting T cell function.

Adoptive Treg therapy has shown promise in reducing GvHD without increasing the risk of disease relapse, highlighting the potential of T cell-based therapy to selectively suppress GvHD while preserving the GvL response [26]. However, the scalability of Treg expansion and the stability of Tregs as immunoregulatory cells pose challenges to the clinical utility of Treg therapy [27]. Additionally, Treg therapy offers limited efficacy in controlling relapse directly. In contrast, the feasibility of scalable DNT production has been demonstrated in phase I clinical trials where an average of 4 × 10^8^ DNTs were expanded from one milliliter of blood used with high purity and sterility [20]. Furthermore, we recently demonstrated the feasibility of combining DNTs obtained from different donors to increase the yield per expansion without compromising the expansion capacity and anti-leukemic activity of DNT [28]. In addition, this study demonstrates that DNTs can effectively improve GvL response or GvHD, depending on the presence or absence of AML. Collectively, these findings support the potential of DNTs as a novel adjuvant therapy to allo-HSCT with the scalability and functionality to be developed as an off-the-shelf therapy for managing both GvHD and disease relapse.

Immune cell functional plasticity in response to the microenvironment allows ACT to be a dynamic therapeutic approach [29, 30]. In our study, we present results demonstrating that DNTs can prevent or treat relapse through their inherent anti-leukemic activity and their ability to promote the GvL effects of Tconv cells in the presence of leukemic cells. Furthermore, we have shown that DNT can mitigate GvHD by impeding the activation and proliferation of alloreactive donor Tconv cells in the absence of leukemia cells and killing proliferating Tconv cells. A major difference between GvHD and GvL are the primary locations where the two phenomena occur. Ni et al*.* reported that increased expression of PD-L1 triggered by elevated levels of IFNγ can cause CD8^+^ Tconv cell exhaustion through PD-L1-PD1 interaction in GvHD-prone tissues while promoting the GvL response of CD8^+^ Tconv cells in lymphoid tissue through PD-L1-CD80 interaction [31]. DNTs are potent producers of IFNγ [15, 17], and can migrate to the BM, the primary site of leukemia engraftment, and GvHD-prone tissues such as the liver [19]. Hence, the type of immune response mediated by DNTs may be dictated by their spatial distribution, where DNTs suppress Tconv cells at the site of GvHD while promoting Tconv cell function at the site of disease relapse.

Given that the xenogeneic model used recapitulates acute GvHD [32], the impact of DNTs on chronic GvHD requires further investigation. Histological analysis revealed severe tissue damage in the lungs of the three mice treated with PBMC + DNT and euthanized on day 52 (Figure S4), whereas no tissue damage was observed in the liver (Figure S10). These findings suggest that DNTs may be more effective at suppressing GvHD in the liver compared to the lungs.

Different studies report positive and negative effects of DNTs on infection and autoimmunity [33]. Thus, it is unknown whether ex vivo expanded DNT therapy would promote or protect allo-HSCT patients from other complications of allo-HSCT. Among the 10 patients who received allogeneic DNT therapy in a phase I clinical trial, none experienced viral or fungal infections, and two patients experienced bacterial infections [20], likely due to preconditioning chemotherapy. The frequency of infection was lower than that seen in patients treated with autologous CAR-T cell therapy after lymphodepleting preconditioning [34]. Further research is needed to deepen our understanding of how DNTs respond in different contexts and to further optimize DNT therapy as a safe and effective adjunct to allo-HSCT.

Acute GvHD is mainly mediated by the interaction between TCR of donor T cells and MHC of the recipient. Given that GvL responses also rely on T-cell function, suppressing GvHD while promoting GvL is challenging. While against the common dogma, TCR-MHC unrestricted anti-tumoral activities of CD8^+^ T cells have been reported [35, 36]. Hence, DNTs may have induced TCR-MHC unrestricted activities of CD8^+^ T cells to promote GvL response without aggrevating GvHD, which requires further investigation.

Donor cell chimerism is an important predictor for allo-HSCT patient outcomes, where poor chimerism is associated with worse patient outcomes. We have previously studied the impact of DNT treatment in human allogeneic hematopoietic stem/progenitor cell (HSPC) engraftment in a xenograft model and demonstrated that DNTs do not affect allogeneic HSPC engraftment nor their differentiation into different hematopoietic lineages [17]. These data are consistent with the safety of allogeneic DNT therapy for allo-HSCT patients in a phase I clinical trial [20] and support that DNTs do not adversely affect the immune reconstitution of allo-HSCs. However, further clinical investigation is needed to elucidate the impact of DNT therapy on the long-term reconstitution and graft stability in allo-HSCT patients.

Clinical observations have revealed that allo-HSCT patients with higher levels of DNTs exhibit a reduced risk of disease relapse and a lower incidence and severity of GvHD [37–39]. Moreover, a recently concluded phase I clinical trial utilizing third-party DNTs for treating relapsed AML following allo-HSCT demonstrated promising outcomes. None of the patients experienced adverse events above grade 2, and complete remission was achieved in 60.0% of the patients without any signs of HvG rejection after receiving multiple DNT infusions [18]. However, the impact of ex vivo expanded allogeneic DNTs on the GvL effect and GvHD, when administered as an adjuvant to allo-HSCT, was unclear. The studies presented here uncover the potential synergy between allogeneic DNT therapy with Tconv cells to mediate potent leukemia control while suppressing GvHD.

Previous studies have reported that endogenous DNTs exhibit varied immune functions depending on the disease context, including both suppressing or inducing autoimmune disease [40], promoting transplant tolerance [41], suppressing GvHD [39], and enhancing anti-cancer activity [33, 40, 42]. Hence, unravelling the endogenous function of DNTs has been challenging. In this study, we demonstrated the multifaceted activities of DNTs that are regulated by their specific environment, i.e. in the presence or absence of leukemic cells. Our findings provide insights into the diverse and, at times, contrasting immune functions associated with endogenous DNTs across different disease conditions.

Mechanistically, we have demonstrated that DNTs mediate anti-tumor activity in an NKG2D, DNAM-1, and TRAIL-dependent manner [16, 17, 43]. Previously, NKG2D on NK cells and TRAIL on Tconv cells were shown to be involved in GvHD suppression [44, 45]. However, blocking of TRAIL or NKG2D had insignificant or only modest effects on DNT-mediated suppression of Tconv cells. Instead, CD18 was involved in DNT-mediated suppression of GvHD-induced by Tconv cells. Given that both GvHD and HvG rejection is driven by the alloreactivity of Tconv cells, this finding provides insights into how DNTs and CAR-DNTs may evade HvG rejection [19, 28] in allogeneic recipients.

CD18 forms heterodimers with CD11a, CD11b, CD11c, and CD11d to form LFA-1 [46], complement receptor (CR) 3 [47], CR4 [47], and integrin αMβ2 [48], respectively. The high co-expression of CD18 and CD11a on DNTs, combined with the upregulation of the LFA-1 ligand ICAM-1 on activated Tconv cells, suggests that DNTs utilize LFA-1 to engage with activated Tconv cells via ICAM-1. Notably, LFA-1 inhibition only reduced DNTs’ ability to suppress CD8^+^ T cell alloreactivity but did not affect their ability to promote the anti-leukemic activity of CD8^+^ T cells, suggesting that LFA-1 is preferentially involved in GvHD-suppressive activity of DNT. In addition to the well-known role of LFA-1 in immune cell migration and immune synapse formation by cytotoxic T or NK cells [49], LFA-1 has also been shown to be involved in CD4^+^ T_reg_ cell-mediated immune suppression [50, 51]. More recent reports revealed the importance of LFA-1 signalling in fine-tuning the effector function of Tconv cells [52, 53]. LFA-1 on DNTs may be involved in the immunoregulatory activity of DNTs by binding to the upregulated ICAM-1 on activated Tconv cells to form the immune synapse needed for the cytotoxic activity of DNTs, sending the downstream signal that evokes the immunosuppressive activity of DNTs, or both. Hence, further investigations are needed to understand the role of LFA-1 in DNT-mediated GvHD suppression.

In the presence of AML cells, DNTs promoted the anti-leukemic activities of Tconv cells by releasing soluble factors, including IFNγ and TNFα. The importance of IFNγ released by Tconv cells in mediating the anti-tumoral activity has been recently reported in the context of CD19-CAR-Tconv cell therapy against B-cell malignancies [54, 55]. Further, we showed that DNT-secreted IFNγ can sensitize AML cells to DNT-mediated cytotoxicity through upregulating NKG2D ligands [17]. The finding from this study demonstrates an important role of IFNγ in inducing the anti-cancer activity of Tconv cells, an underappreciated role of IFNγ in ACTs [17].

In addition to *IFNG* and *TNFA,* DNTs expressed significantly higher levels of chemokine genes like CCL3, CCL4, and CCL5 than Tconv cells. Although blocking the activity of each of these secreted proteins had no effect on the ability of DNTs to promote the anti-leukemic activity of Tconv cells in vitro*,* several studies have reported the role of CCL3, CCL4, and CCL5 for the recruitment of effector Tconv cells to cancer sites to promote anti-cancer activities [56–58]. Therefore, while DNTs relied on IFNγ and TNFα to promote the anti-leukemic activity of Tconv cells, they may also promote recruitment of Tconv cells to the site of leukemia engraftment in vivo via the release of chemokines.

AML is a highly heterogeneous disease, contributing to its resistance to various treatment approaches [59]. In previous studies, about 20% of AML samples tested were resistant to DNTs [17]. DNTs demonstrated superior cytotoxicity against AML blasts obtained from patients with higher bone marrow (BM) AML counts and blasts classified as M5 according to the FAB classification [17]. However, AML blasts from favorable, intermediate, and high-risk patients displayed similar susceptibility to DNTs [17]. Using genome-wide CRISPR screening, we identified that AML cells lacking FCGR1A expression or exhibiting high levels of the Spt-Ada-Gcn5-Acetyltransferase (SAGA) complex, were particularly resistant to DNTs, which may be utilized as markers to identify patients who may benefit more from DNT therapy. Further investigation is needed to determine whether the synergy between DNTs and Tconv cells is influenced by specific AML subtypes or by particular molecular pathway.

Our previous work demonstrated that chemotherapy can sensitize AML cells to DNTs by upregulating NKG2D and DNAM-1 ligands recognized by DNT [16]. Additionally, DNTs can synergize with the Bcl-2 inhibitor Venetoclax and the hypomethylating agent azacytidine to elicit superior anti-leukemic activity [60]. In addition to the previous findings, this study supports combining DNTs with allo-HSCT to yield a synergistic anti-leukemic effect, potentially achieving deeper remission in patients with a heterogeneous disease like AML.

Collectively, the findings from this study and the nature of off-the-shelf cell therapy of DNTs support the use of allogeneic DNT therapy as an adjuvant to allo-HSCT, which has the potential to address both disease relapse and GvHD.

## Materials and methods

### Sex as a biological variable

Our study examined male and female DNT and PBMC healthy donors and AML patients, and similar findings are reported for both sexes. For xenograft model, female mice were used to ensure efficient engraftment of AML cells. It is unknown whether the findings are relevant for male mice.

### Ex vivo expansion of DNTs

DNT expansions were performed as previously described [19]. Briefly, 60–80 millilitres of peripheral blood was obtained from healthy donors, and CD4^+^ and CD8^+^ cells were depleted from peripheral blood mononuclear cell (PBMC) during density-gradient centrifugation using CD4- and CD8-depletion kits (SepMate™; STEMCELL l Technology). CD4^+^ and CD8^+^-depleted PBMCs were expanded on anti-CD3 antibody-coated plates (OKT3; Biolegend) for 3 days in AIM-V media (ThermoFisher) with 250 IU/ml of IL-2 (Proleukin, Novartis Pharmaceuticals, Canada); soluble anti-CD3 antibody and IL-2 were added to the cultures every 2–4 days. The purity of DNTs was assessed on days 0, 10, and 14 of the expansion by staining cells with fluorochrome-conjugated anti-human CD3, -CD4, -CD8, and -CD56 antibodies and flow cytometry analysis. DNTs were used between days 10 and 20 of culture.

### Flow cytometry-based in vitro killing assay

DNTs or CD8^+^ conventional T (Tconv) cells were co-cultured with AML cells for 2–4 h. Subsequently, cells were stained with anti-human CD3, CD33, and Annexin V and analyzed using flow cytometry. Specific killing was calculated by:$\frac{{{\% AnnexinV}^{+}}_{with\ T\ cell}-{{\% AnnexinV}^{+}}_{without\ T\ cell}}{{{100-\% AnnexinV}^{+}}_{without\ T\ cell}} \times 100$. For assessing the cytotoxicity of DNTs against activated Tconv cells, PBMCs were stained with CFSE (1 µM) followed by stimulation with anti-CD3/CD28 activation beads (Thermofisher Scientifics) for 3 days. Subsequently, the activated PBMCs were co-incubated with DNT cells for 4-h. The cultures were stained with CD3, CD4, CD8, and Annexin V, and % specific killing of the activated Tconv cells was determined by:$\frac{{{\% AnnexinV}^{+}}_{with\ DNT}-{{\% AnnexinV}^{+}}_{without\ DNT}}{{{100-\% AnnexinV}^{+}}_{without\ DNT}} \times 100$

### Mixed lymphocyte reaction (MLR)

MLR assay was set up by co-culturing CFSE-labelled (1 µM) PBMC with irradiated (3000 cGy) allogeneic PBMCs at a 2:1 ratio for 4–6 days in the presence or absence of ex vivo expanded third-party DNTs. The degree of alloreactive Tconv cell proliferation was determined by flow cytometry. To determine allo-reactivity, activated CD8^+^ T cells were isolated from the MLR assay using a CD8-positive selection kit (StemCell Tech.) and used as effector cells agasint fresh allogeneic PBMCs. After 4 h of coculture, the viability of the target cells was determined by Annexin V-staining and flow cytometry. For blocking assays, neutralizing antibodies or corresponding isotype controls (10 µg/ml or as indicated) were added during the MLR or cytotoxicity assay.

### RNA sequencing

DNTs or Tconv cells were obtained from 3 healthy donors and ex vivo expanded for 10 days. Total RNA was extracted from 10^7^ expanded DNT cells using RNeasy Plus Mini Kit (Qiagen) following the manufacturer’s instruction. Briefly, cells were harvested and lysed, and equal volume of 70% ethanol was added. The lysates were immediately transferred to RNeasy Mini Spin Column. DNase I digestion was performed on column for 15 min at room temperature. Eluted RNA was assessed for quality by O.D. 260/280 ratios greater than 2.0. Total RNA was converted to cDNA using M-MLV reverse transcriptase. RNA-seq libraries were prepared using the SMARTer Stranded Total RNA-Seq Kit v2—Pico Input Mammalian (634418, Takara). The quality of the library was verified using TapeStation (Tape 2200, Agilent Technologies) and 150-bp paired-end sequencing was performed by GeneSeeq Technology Inc. (Toronto, Ontario) with ~ 80 million reads per sample.

### Xenograft models

For all xenograft experiments, 6–10 weeks-old NOD. Cg-*Prkdc*^*scid*^* Il2rg*^*tm1Wjl*^/SzJ (NSG) mice (Jackson Laboratories, Bar Harbor, ME) maintained at the University Health Network (UHN) animal facility were used. NSG mice were sublethally irradiated (250 cGy) 24 h prior to use. Naïve mice or mice infused with MV4-11 (2 × 10^6^ cells per mouse infused intravenously) or primary AML patient samples (5 × 10^6^ per mouse infused intravenously) were intravenously injected with PBS or 2–5 × 10^6^ healthy donor-derived PBMCs 3 days post-AML infusion with or without 2 × 10^7^ DNTs infused on days 6, 9, and 12 post-AML infusion. For the GvHD-xenograft model, mouse body weight change, general condition and survival were monitored. % body weight change was calculated as $\frac{{body\ weight}_{day 0}- {body\ weight}_{day x}}{{body\ weight}_{day 0}} \times 100$. Mice with weight loss greater than 20% were euthanized. For histological analysis, the liver and lungs were obtained, fixed in 10% buffered Formalin for 24–48 h, and then in 70% ethanol until samples were sent for hematoxylin and eosin histological analysis. Histological staining and picture acquisition was done by the Advanced Optical Microscopy Facility at UHN. For AML xenograft models, mice were sacrificed two weeks after the last DNT injection, and the frequency of AML cells in the bone marrow was determined using flow cytometry as described previously [17, 19]. For CD18KO and AML-encountered DNT (AE-DNT) experiments, naïve-NSG mice were intravenously infused with 2 × 10^6^ PBMCs. Subsequently, the mice were treated with PBS, 1.5 × 10^7 CD18KO^DNT or 1.5 × 10^7 scr^DNT on days 3 and 6 post PBMC-injection or with 10^7^ AE-DNT or DNT on day 3 post-PBMC infusion. In all experiments, rIL-2 (Proleukin) was administered (10^4^ IU/mouse) intravenously at the time of DNT infusion.

### Generation of CD18 knock-out DNTs

DNTs cells on day 3 of culture were nucleofected with RNP complex assembled with a two-component guide RNA (gRNA). CD18-specific CRISPR RNA (crRNA; Exon 5, position 44903503: 5’ CGTTCAACGTGACCTTCCGG 3’) and trans-activating CRISPR RNA (tracrRNA) along with negative Control crRNA #1 (scrambled crRNA) were obtained from Integrated DNA Technologies (IDT) and assembled using IDT protocol. In brief, tracrRNA and crRNAs dissolved in Nuclease-free Duplex buffer were mixed 1:1 to a final concentration of 100 μM, incubated at 95 °C for 5 min and slowly cooled to RT. RNP complexes were prepared just before nucleofection using Cas9 nuclease V3 (IDT) and tracrRNA/crRNA duplex diluted in PBS to a total volume of 5μl. Nucleofection was carried out with P3 Primary Cell 4D-Nucleofector X Kit S (Lonza) according to the manufacturer’s protocol using the Amaxa Nucleofector system (Lonza). Briefly, DNTs were mixed with 5μl of the RNP mixture and nucleofected using Voltage DS130. Knockdown of cell surface CD18 expression was measured by Flow Cytometry using an antibody specific to CD18 (APC anti-human CD18 clone 1B4/CD18; Biolegend). CD18 surface expression from cells nucleofected with negative control crRNA served as a control.

### Assessing the impact of DNTs on the anti-leukemic activity of Tconv cells

PBMCs were co-cultured alone or with AML cells, MV4-11 or OCI-AML3, in the presence or absence of DNTs for 5 days. Subsequently, CD8^+^ T cells were isolated using a CD8^+^ isolation kit (STEMCELL Technologies) and used as effector cells against the AML cell line used for the initial co-culture in a subsequent cytotoxicity assay**.** To assess the impact of DNT-secreted soluble factors on the anti-leukemic activity of CD8^+^ T cells, CD8^+^ T cells or MV4-11 cells were cultured in supernatants obtained from DNT alone or DNT + MV4-11 cultures (^DNT^supernatant and ^DNT+AML^supernatant, respectively) for overnight. Subsequently, AML and CD8^+^ T cells were washed and used in an in vitro cytotoxicity assay at 1:1 CD8^+^ T cell: AML ratio, incubated for 4 h.

### Human samples and study approval

Human blood and bone marrow cells were collected from healthy adult donors and AML patients after obtaining written informed consent and used according to UHN Research Ethics Board (05-0221-T) approved protocols. PBMCs from healthy donors or AML patients were separated by Ficoll (GE Healthcare) density gradient. AML patient samples were viably frozen in the Princess Margaret Leukemia Bank and stored in liquid nitrogen until use. Animal studies were approved by the UHN Animal Care Committee (Permit Number: 741.22) and carried out in accordance with the Canadian Council on Animal Care Guidelines.

### Statistical analysis

All graphs and statistical analyses were generated using GraphPad Prism 5. Student’s *t-test*, one-way ANOVA test, two-way ANOVA test, and Gehan-Breslow-Wilcoxon test were used. *p < 0.05; **p < 0.01; ***p < 0.001; ****p < 0.0001 indicate significance between experimental and control values. Error bars represent ± SD.

## Supplementary Information

Supplementary Material 1.

Supplementary Material 2.

Supplementary Material 3.

## Data Availability

The datasets used and/or analyzed during the current study are available from the corresponding author upon reasonable request.
