# Supplementary material for: Allogeneic DNT cell therapy synergizes with T cells to promote anti-leukemic activities while suppressing GvHD
Source: J Exp Clin Cancer Res. 2025 Jan 28;44:28. doi: 10.1186/s13046-024-03247-w (PMC11773727; doi:10.1186/s13046-024-03247-w)
Supplement: Supplementary file 1 — Supplementary Material 1. [file 13046_2024_3247_MOESM1_ESM.pptx]

## Slide 1
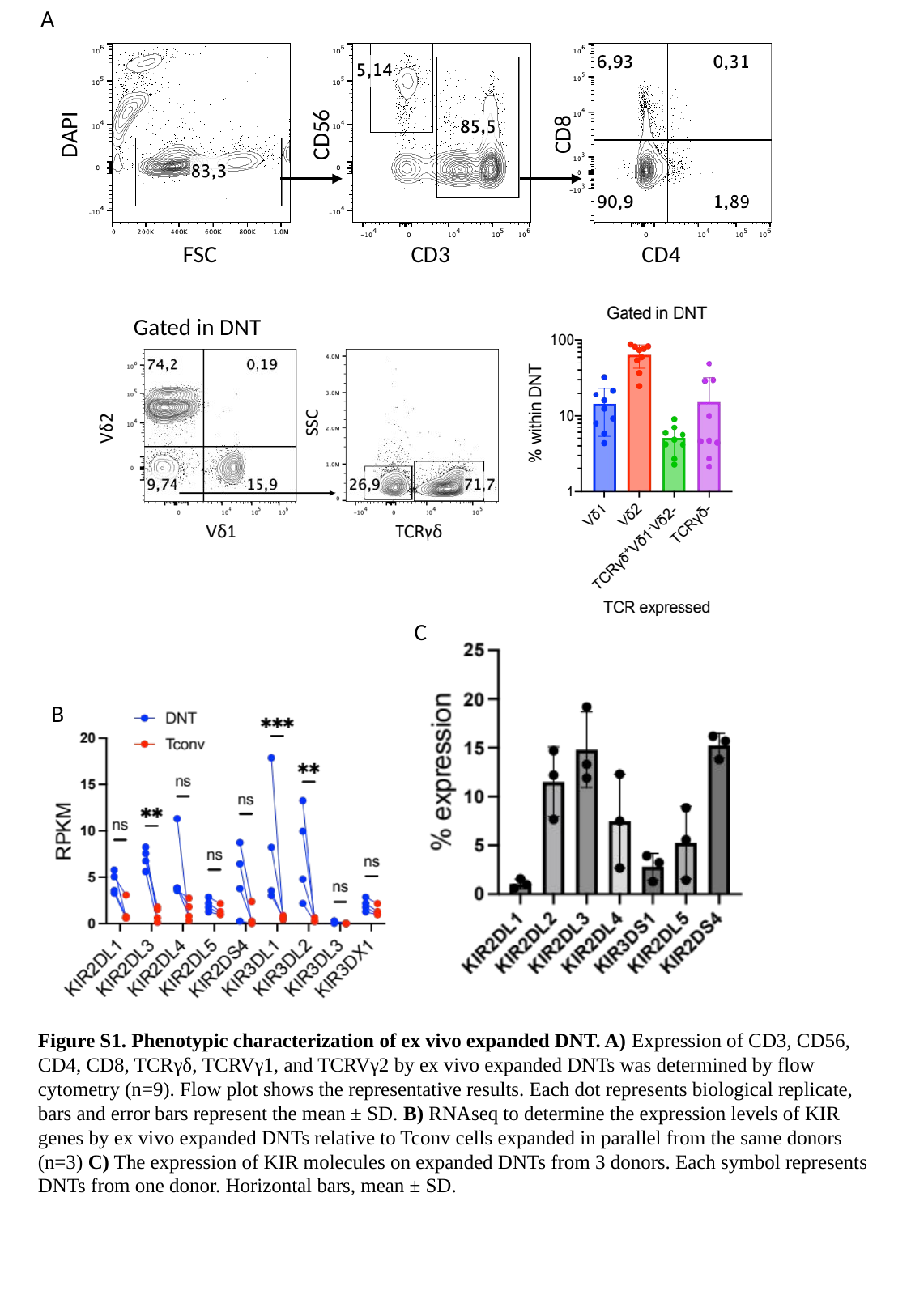

A
DAPI
CD56
CD8
FSC
CD3
CD4
Gated in DNT
C
B
Figure S1. Phenotypic characterization of ex vivo expanded DNT. A) Expression of CD3, CD56, CD4, CD8, TCRγδ, TCRVγ1, and TCRVγ2 by ex vivo expanded DNTs was determined by flow cytometry (n=9). Flow plot shows the representative results. Each dot represents biological replicate, bars and error bars represent the mean ± SD. B) RNAseq to determine the expression levels of KIR genes by ex vivo expanded DNTs relative to Tconv cells expanded in parallel from the same donors (n=3) C) The expression of KIR molecules on expanded DNTs from 3 donors. Each symbol represents DNTs from one donor. Horizontal bars, mean ± SD.

## Slide 2
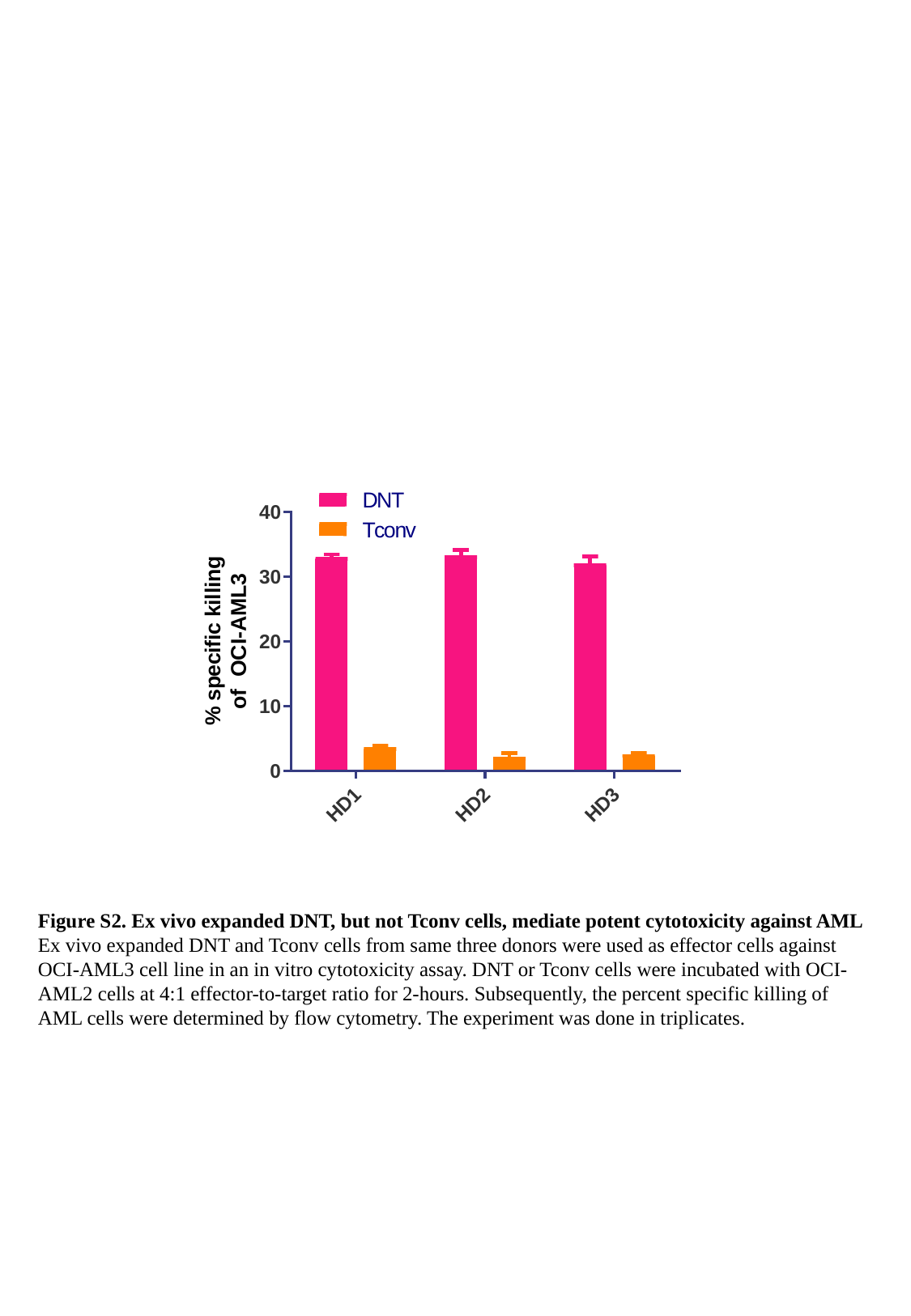

Figure S2. Ex vivo expanded DNT, but not Tconv cells, mediate potent cytotoxicity against AML Ex vivo expanded DNT and Tconv cells from same three donors were used as effector cells against OCI-AML3 cell line in an in vitro cytotoxicity assay. DNT or Tconv cells were incubated with OCI-AML2 cells at 4:1 effector-to-target ratio for 2-hours. Subsequently, the percent specific killing of AML cells were determined by flow cytometry. The experiment was done in triplicates.

## Slide 3
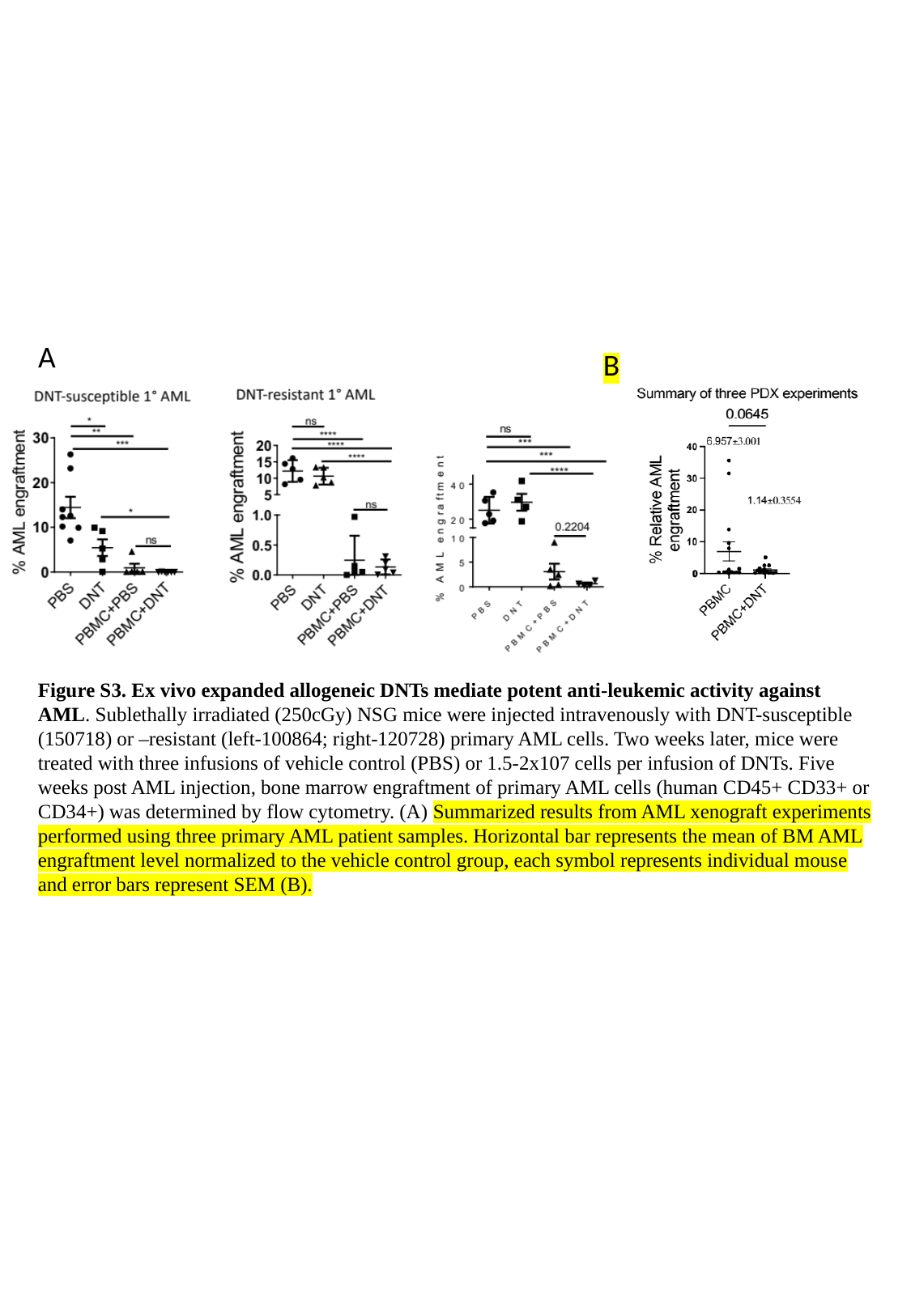

A
B
Figure S3. Ex vivo expanded allogeneic DNTs mediate potent anti-leukemic activity against AML. Sublethally irradiated (250cGy) NSG mice were injected intravenously with DNT-susceptible (150718) or –resistant (left-100864; right-120728) primary AML cells. Two weeks later, mice were treated with three infusions of vehicle control (PBS) or 1.5-2x10^7^ cells per infusion of DNTs. Five weeks post AML injection, bone marrow engraftment of primary AML cells (human CD45+ CD33+ or CD34+) was determined by flow cytometry. (A) Summarized results from AML xenograft experiments performed using three primary AML patient samples. Horizontal bar represents the mean of BM AML engraftment level normalized to the vehicle control group, each symbol represents individual mouse and error bars represent SEM (B).

## Slide 4
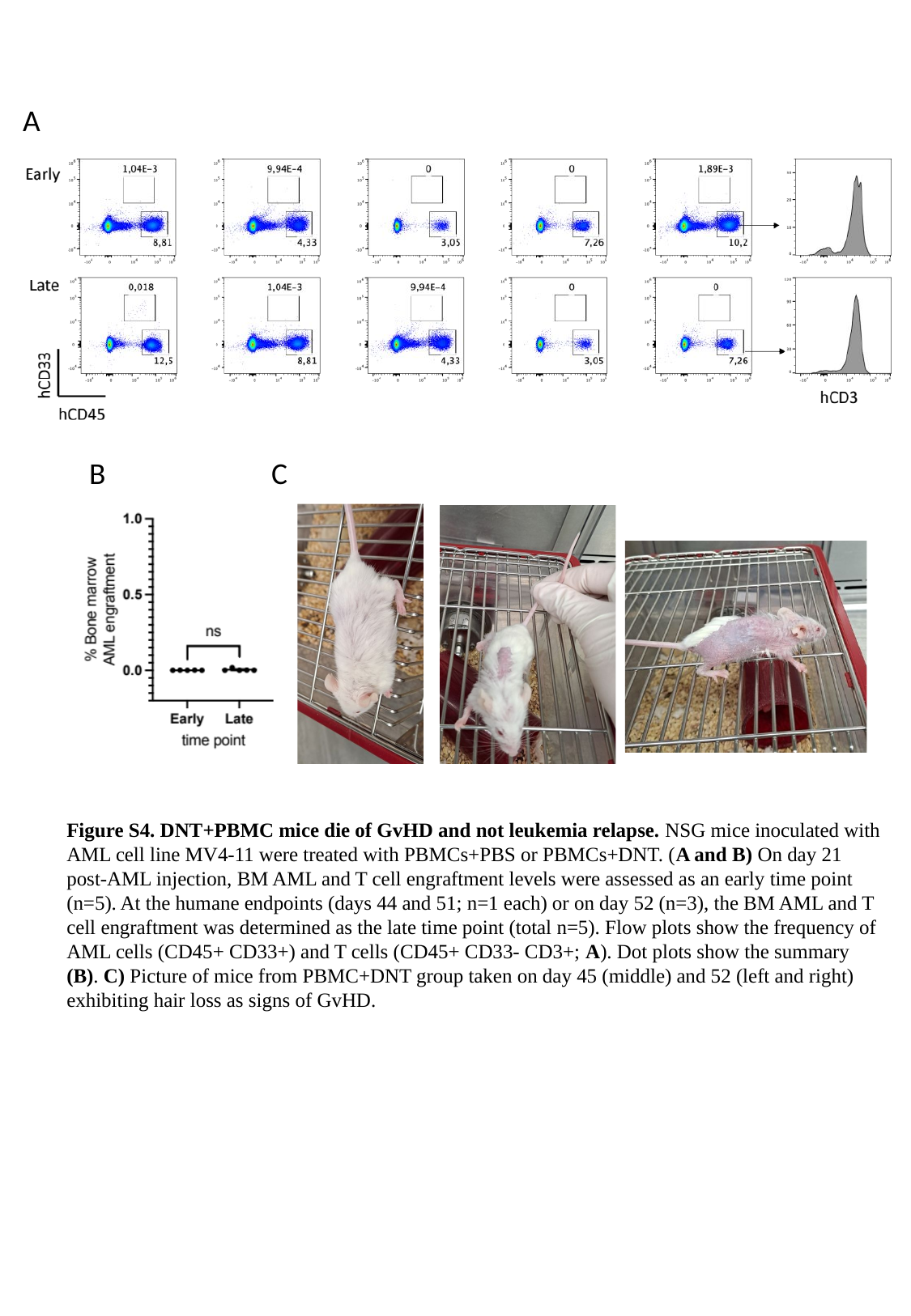

A
B
C
Figure S4. DNT+PBMC mice die of GvHD and not leukemia relapse. NSG mice inoculated with AML cell line MV4-11 were treated with PBMCs+PBS or PBMCs+DNT. (A and B) On day 21 post-AML injection, BM AML and T cell engraftment levels were assessed as an early time point (n=5). At the humane endpoints (days 44 and 51; n=1 each) or on day 52 (n=3), the BM AML and T cell engraftment was determined as the late time point (total n=5). Flow plots show the frequency of AML cells (CD45+ CD33+) and T cells (CD45+ CD33- CD3+; A). Dot plots show the summary (B). C) Picture of mice from PBMC+DNT group taken on day 45 (middle) and 52 (left and right) exhibiting hair loss as signs of GvHD.

## Slide 5
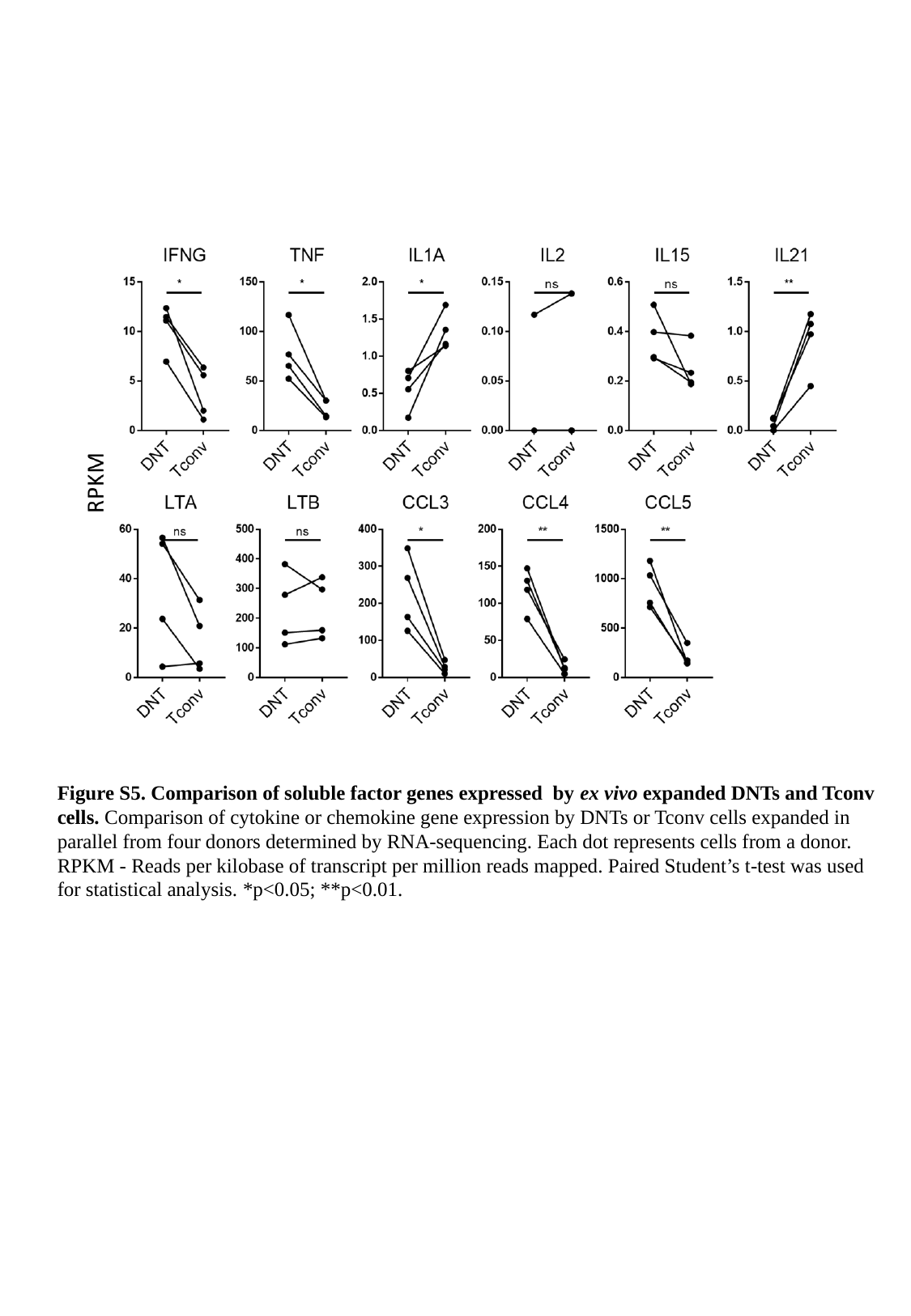

Figure S5. Comparison of soluble factor genes expressed by ex vivo expanded DNTs and Tconv cells. Comparison of cytokine or chemokine gene expression by DNTs or Tconv cells expanded in parallel from four donors determined by RNA-sequencing. Each dot represents cells from a donor. RPKM - Reads per kilobase of transcript per million reads mapped. Paired Student’s t-test was used for statistical analysis. *p<0.05; **p<0.01.

## Slide 6
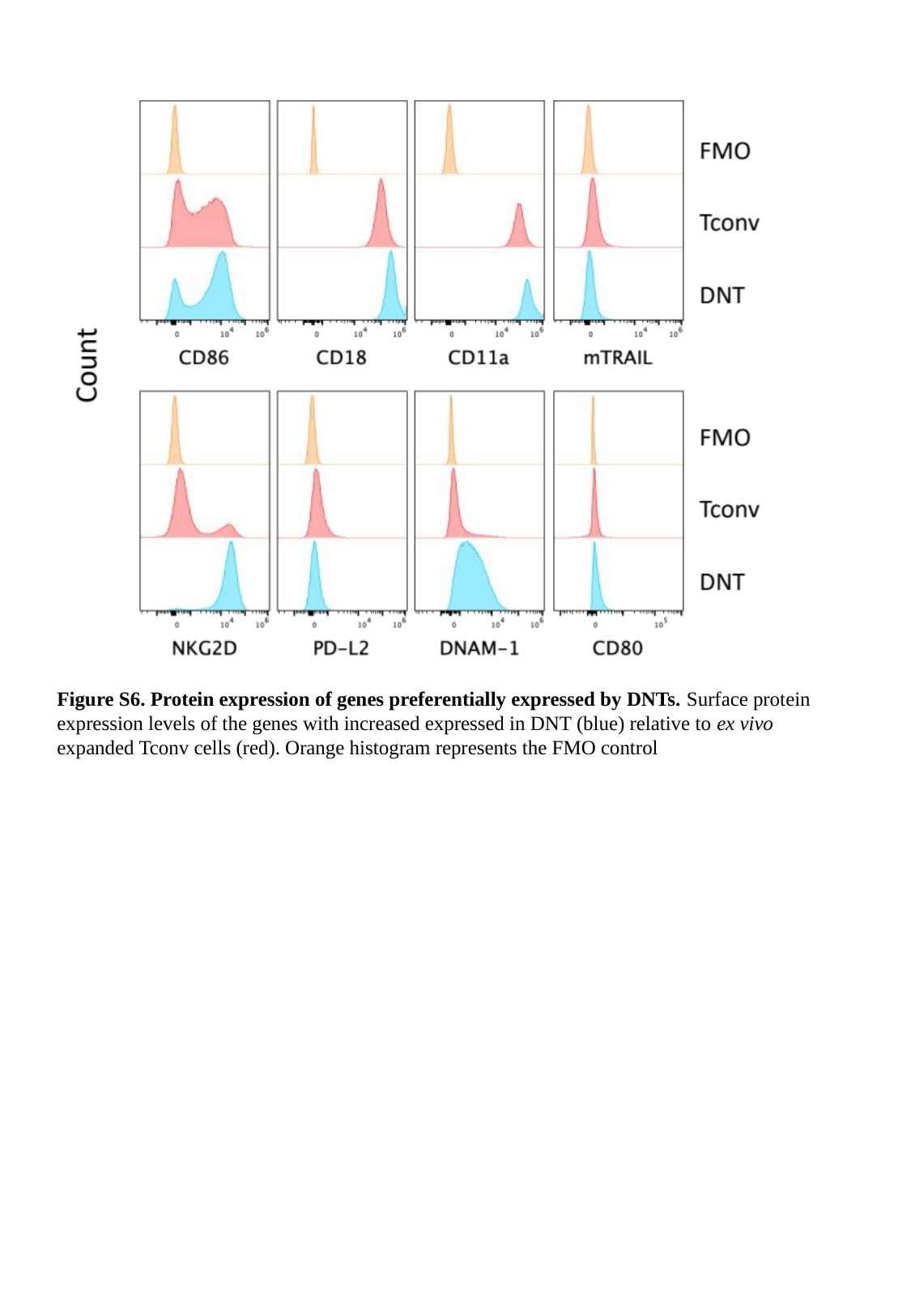

Figure S6. Protein expression of genes preferentially expressed by DNTs. Surface protein expression levels of the genes with increased expressed in DNT (blue) relative to ex vivo expanded Tconv cells (red). Orange histogram represents the FMO control

## Slide 7
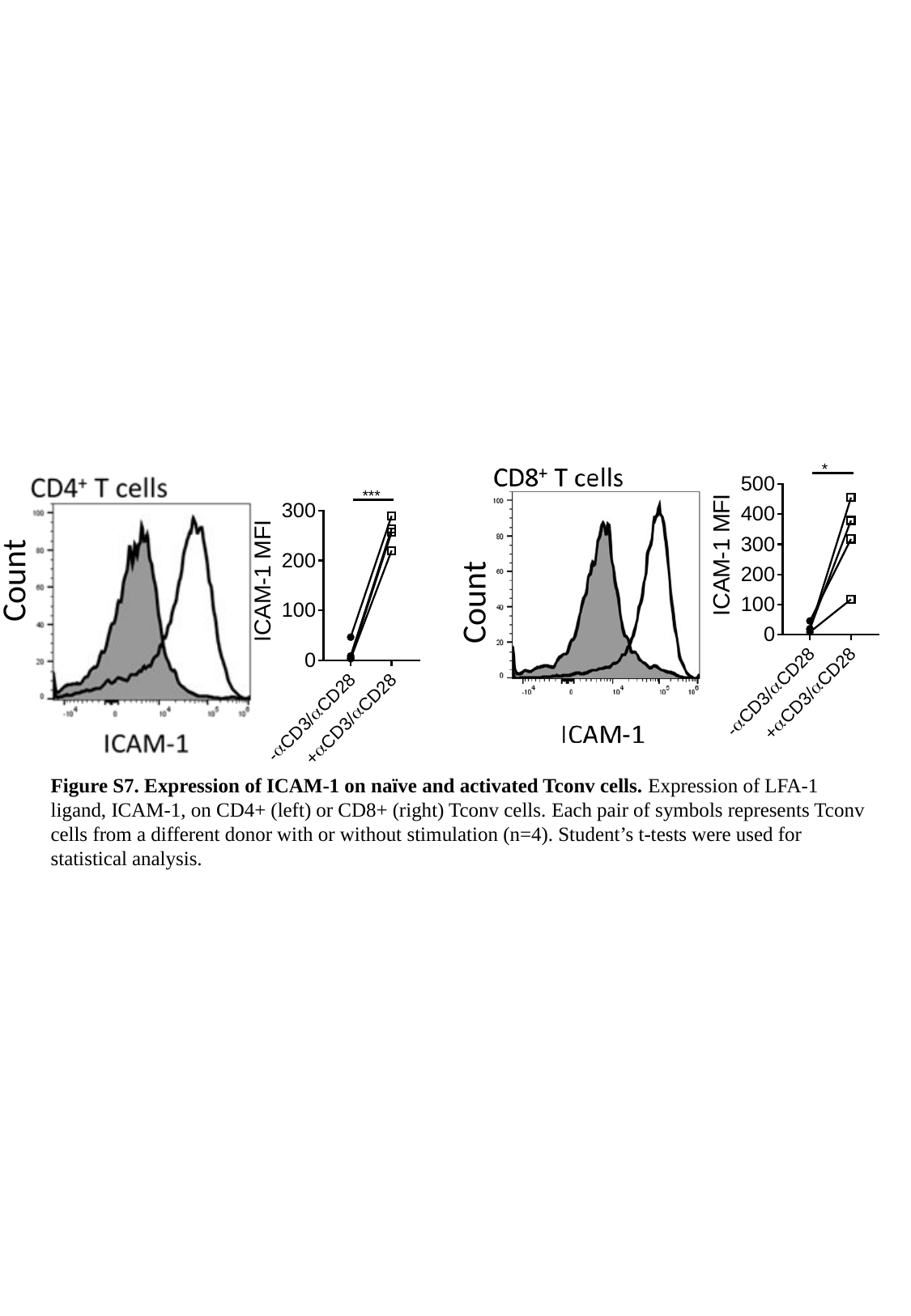

Count
Count
Figure S7. Expression of ICAM-1 on naïve and activated Tconv cells. Expression of LFA-1 ligand, ICAM-1, on CD4+ (left) or CD8+ (right) Tconv cells. Each pair of symbols represents Tconv cells from a different donor with or without stimulation (n=4). Student’s t-tests were used for statistical analysis.

## Slide 8
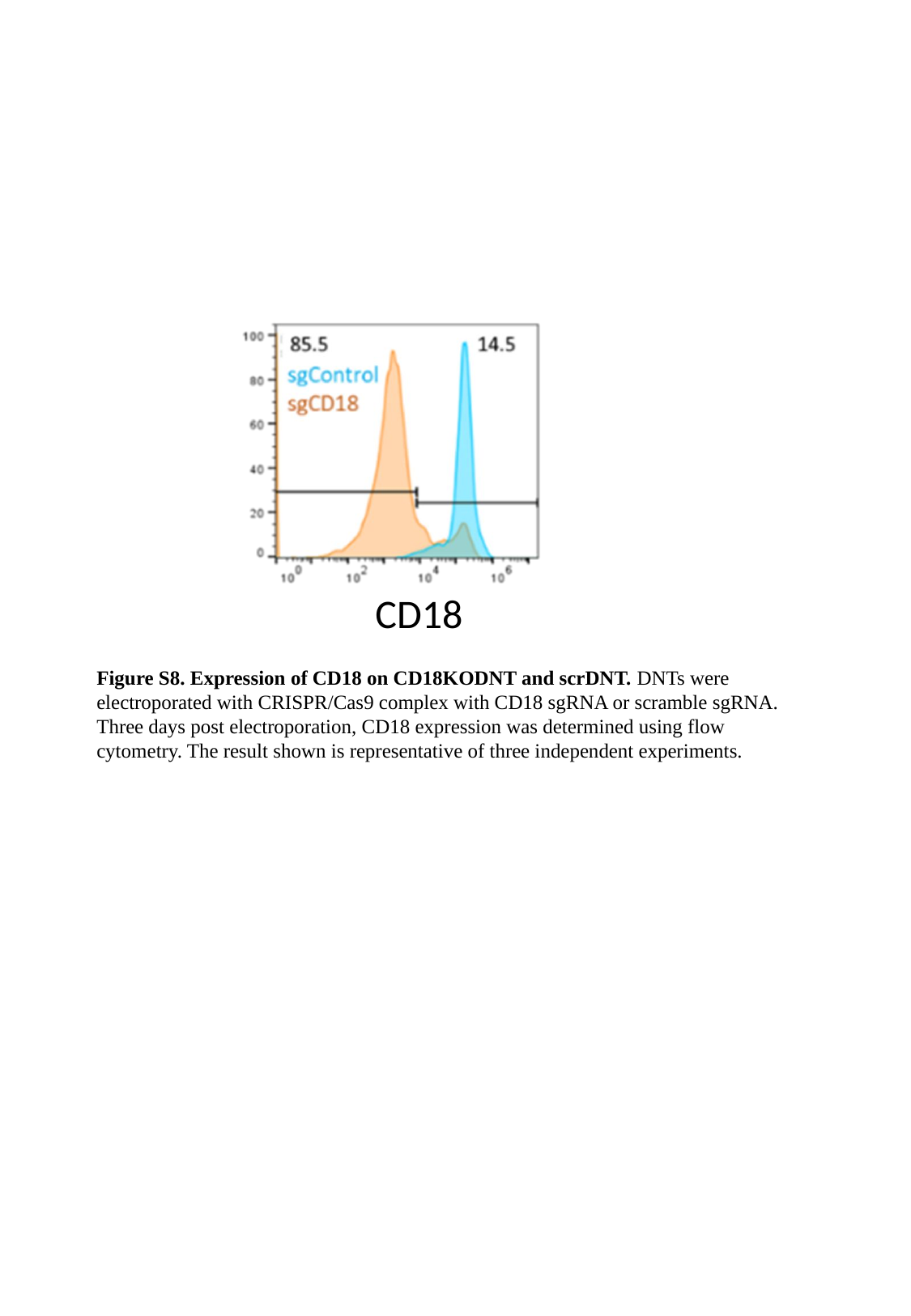

CD18
Figure S8. Expression of CD18 on CD18KODNT and scrDNT. DNTs were electroporated with CRISPR/Cas9 complex with CD18 sgRNA or scramble sgRNA. Three days post electroporation, CD18 expression was determined using flow cytometry. The result shown is representative of three independent experiments.

## Slide 9
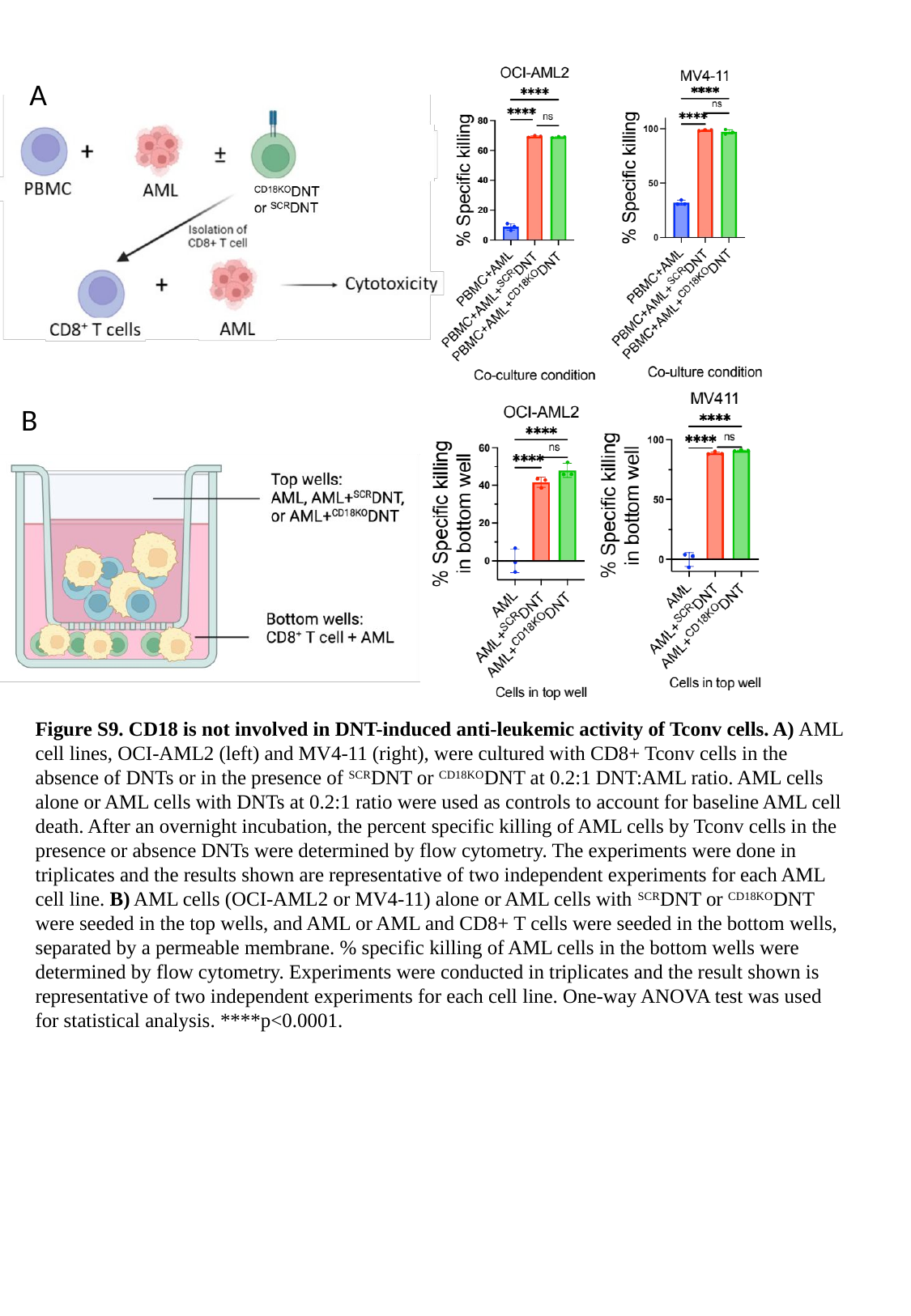

A
B
Figure S9. CD18 is not involved in DNT-induced anti-leukemic activity of Tconv cells. A) AML cell lines, OCI-AML2 (left) and MV4-11 (right), were cultured with CD8+ Tconv cells in the absence of DNTs or in the presence of SCRDNT or CD18KODNT at 0.2:1 DNT:AML ratio. AML cells alone or AML cells with DNTs at 0.2:1 ratio were used as controls to account for baseline AML cell death. After an overnight incubation, the percent specific killing of AML cells by Tconv cells in the presence or absence DNTs were determined by flow cytometry. The experiments were done in triplicates and the results shown are representative of two independent experiments for each AML cell line. B) AML cells (OCI-AML2 or MV4-11) alone or AML cells with SCRDNT or CD18KODNT were seeded in the top wells, and AML or AML and CD8+ T cells were seeded in the bottom wells, separated by a permeable membrane. % specific killing of AML cells in the bottom wells were determined by flow cytometry. Experiments were conducted in triplicates and the result shown is representative of two independent experiments for each cell line. One-way ANOVA test was used for statistical analysis. ****p<0.0001.

## Slide 10
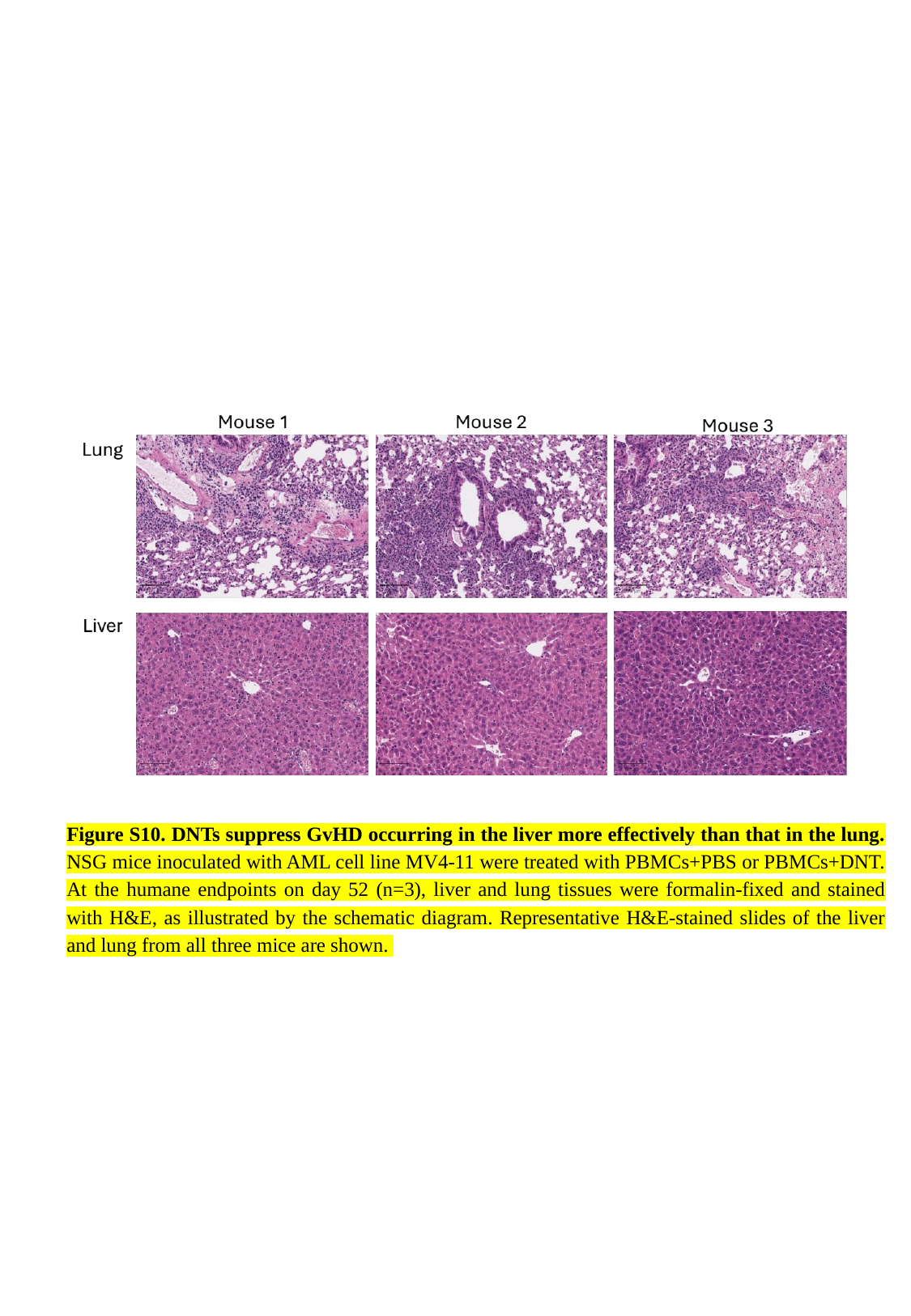

Figure S10. DNTs suppress GvHD occurring in the liver more effectively than that in the lung. NSG mice inoculated with AML cell line MV4-11 were treated with PBMCs+PBS or PBMCs+DNT. At the humane endpoints on day 52 (n=3), liver and lung tissues were formalin-fixed and stained with H&E, as illustrated by the schematic diagram. Representative H&E-stained slides of the liver and lung from all three mice are shown.
