# Supplementary material for: Allogeneic DNT cell therapy synergizes with T cells to promote anti-leukemic activities while suppressing GvHD
Source: J Exp Clin Cancer Res. 2025 Jan 28;44:28. doi: 10.1186/s13046-024-03247-w (PMC11773727; doi:10.1186/s13046-024-03247-w)
Supplement: Supplementary file 2 — Supplementary Material 2. [file 13046_2024_3247_MOESM2_ESM.pdf]

Supplementary materials and methods

GvHD-model tissue damage scoring

Mice treated with PBS, DNT, or PBMC were sacrificed and liver and lung tissues harvested, fixed in 10% buffered formalin, and H&E stained. Liver and lung histology slides were blindly scored by a pathologist following the scoring charts below:

Liver GvHD scoring

	0	1	2	3
Portal inflammation	Absent	**Mild** Present in fewer than 30% of portal tracts	**Moderate** Present in more 30%-50% of portal tracts	**Severe** Present in majority (more than 50%) of portal tracts
Lobular Inflammation	Absent	**Mild** With little hepatocytes necrosis or apoptosis	**Moderate** With focal necrosis causing confluence and/or several apoptosis	**Severe** With bridging/severe parenchymal necrosis
Bile duct injury	Absent	**Mild** Slight duct epithelium disorder with cytoplasmic eosinophilia	**Moderate** Duct epithelial disorganization with partial necrosis of affected ducts	**Severe** Extensive duct epithelial disorganization with complete necrosis of affected ducts
Bile duct loss	Absent	**Mild** Affecting 30% or less	**Moderate** Affecting 30-60%	**Severe** Affecting more than 60%
Cholestasis	Absent	**Mild** Visible only at 20X or higher magnification	**Moderate** Visible at 5-10x magnification but not easily	**Severe** Visible easily at 5x magnification
Total Score (/15)				

Lung GvHD Scoring

	0	1	2	3
inflammation	Absent	**Mild** Present around vessels without endotheliitis	**Moderate** Present with endotheliitis +- / septal expansion+/- mild alveolar extension	**Severe** Present with extensive septal expansion, heavy intra-alveolar inflammation +/- hemorrhages +/- alveolar involvement
Bronchial/bronchiolar Epithelial injury	Absent	**Mild** Subepithelial inflammation with no intra-epithelial inflammation	**Moderate** Subepithelial inflammation with intra-epithelial inflammation but no epithelial necrosis and/or apoptosis	**Severe** Subepithelial inflammation with intra-epithelial inflammation and necrosis
Total Score (/6)				

Antibodies, flow cytometry and ELISA

The following anti-human antibodies were used for cell staining: CD3 (HIT3a)-FITC or -PECy7, CD4 (RPA-T4)-FITC or -PE, CD8 (SK1)-FITC or -PE, CD34 (581)-FITC or -PE, CD33 (WM53)-APC or –PECy5, and CD28 (CD28.2)-APC, OX40 (Ber-ACT35)-APCCy7, ICOS (C398.4A)-PECy7, ICAM-1 (HA58)-FITC, NKG2D (1D11)-PE or purified, TRAIL (RIK-2)-PE, PD-L2 (24F.10C12)-PE, CD18 (1B4/CD18)-PE or purified, CD11a (HI111)-PE, CD80 (2D10)-purified, CD86 (BU63)-PE or purified, PD-1(A17188B)- purified, Annexin V-FITC or -Pacific Blue were purchased from BioLegend. Data acquisitions were performed using either Attune NXT Flow cytometry (Thermofisher Scientific) or LSRII (BD Biosciences). Data were analyzed using FlowJo software (Tree Star, Inc.).

Generation of CD18 knock-out DNTs

DNTs cells on day 3 of culture were nucleofected with RNP complex assembled with a two-component guide RNA (gRNA). CD18-specific CRISPR RNA (crRNA; Exon 5, position 44903503: 5' CGTTCAACGTGACCTTCCGG 3') and trans-activating CRISPR RNA (tracrRNA) along with negative Control crRNA #1 (scrambled crRNA) were obtained from Integrated DNA Technologies (IDT) and assembled using IDT protocol. In brief, tracrRNA and crRNAs dissolved in Nuclease-free Duplex buffer were mixed 1:1 to a final concentration of 100 μM, incubated at 95°C for 5 min and slowly cooled to RT. RNP complexes were prepared just before nucleofection using Cas9 nuclease V3 (IDT) and tracrRNA/crRNA duplex diluted in PBS to a total volume of 5μl. Nucleofection was carried out with P3 Primary Cell 4D-Nucleofector X Kit S (Lonza) according to the manufacturer's protocol using the Amaxa Nucleofector system (Lonza). Briefly, DNTs were mixed with 5μl of the RNP mixture and nucleofected using Voltage DS130. Knockdown of cell surface CD18 expression was measured by Flow Cytometry using antibody specific to CD18 (APC anti-human CD18 clone 1B4/CD18; Biolegend). CD18 surface expression from cells nucleofected with Negative control crRNA served as a control.
